# The Concise Guide to PHARMACOLOGY 2015/16: Catalytic receptors

**DOI:** 10.1111/bph.13353

**Published:** 2015-12-09

**Authors:** Stephen PH Alexander, Doriano Fabbro, Eamonn Kelly, Neil Marrion, John A Peters, Helen E Benson, Elena Faccenda, Adam J Pawson, Joanna L Sharman, Christopher Southan, Jamie A Davies, CGTP Collaborators

**Affiliations:** ^1^ School of Biomedical Sciences University of Nottingham Medical School Nottingham NG7 2UH UK; ^2^ PIQUR Therapeutics Basel 4057 Switzerland; ^3^ School of Physiology and Pharmacology University of Bristol Bristol BS8 1TD UK; ^4^ Neuroscience Division, Medical Education Institute, Ninewells Hospital and Medical School University of Dundee Dundee DD1 9SY UK; ^5^ University of Edinburgh Edinburgh EH8 9XD UK

## Abstract

The Concise Guide to PHARMACOLOGY 2015/16 provides concise overviews of the key properties of over 1750 human drug targets with their pharmacology, plus links to an open access knowledgebase of drug targets and their ligands (www.guidetopharmacology.org), which provides more detailed views of target and ligand properties. The full contents can be found at http://onlinelibrary.wiley.com/doi/10.1111/bph.13353/full. G protein‐coupled receptors are one of the eight major pharmacological targets into which the Guide is divided, with the others being: G protein‐coupled receptors, ligand‐gated ion channels, voltage‐gated ion channels, other ion channels, nuclear hormone receptors, enzymes and transporters. These are presented with nomenclature guidance and summary information on the best available pharmacological tools, alongside key references and suggestions for further reading. The Concise Guide is published in landscape format in order to facilitate comparison of related targets. It is a condensed version of material contemporary to late 2015, which is presented in greater detail and constantly updated on the website www.guidetopharmacology.org, superseding data presented in the previous Guides to Receptors & Channels and the Concise Guide to PHARMACOLOGY 2013/14. It is produced in conjunction with NC‐IUPHAR and provides the official IUPHAR classification and nomenclature for human drug targets, where appropriate. It consolidates information previously curated and displayed separately in IUPHAR‐DB and GRAC and provides a permanent, citable, point‐in‐time record that will survive database updates.

## Conflict of interest

The authors state that there are no conflicts of interest to declare.

## Overview

Catalytic receptors are cell‐surface proteins, usually dimeric in nature, which encompass ligand binding and functional domains in one polypeptide chain. The ligand binding domain is placed on the extracellular surface of the plasma membrane and separated from the functional domain by a single transmembrane‐spanning domain of 20‐25 hydrophobic amino acids. The functional domain on the intracellular face of the plasma membrane has catalytic activity, or interacts with particular enzymes, giving the superfamily of receptors its name. Endogenous agonists of the catalytic receptor superfamily are peptides or proteins, the binding of which may induce dimerization of the receptor, which is the functional version of the receptor.

Amongst the catalytic receptors, particular subfamilies may be readily identified dependent on the function of the enzymatic portion of the receptor. The smallest group is the particulate guanylyl cyclases of the natriuretic peptide receptor family. The most widely recognized group is probably the receptor tyrosine kinase (RTK) family, epitomized by the neurotrophin receptor family, where a crucial initial step is the activation of a signalling cascade by autophosphorylation of the receptor on intracellular tyrosine residue(s) catalyzed by enzyme activity intrinsic to the receptor. A third group is the extrinsic protein tyrosine kinase receptors, where the catalytic activity resides in a separate protein from the binding site. Examples of this group include the GDNF and ErbB receptor families, where one, catalytically silent, member of the heterodimer is activated upon binding the ligand, causing the second member of the heterodimer, lacking ligand binding capacity, to initiate signaling through tyrosine phosphorylation. A fourth group, the receptor threonine/serine kinase (RTSK) family, exemplified by TGF‐*β* and BMP receptors, has intrinsic serine/threonine protein kinase activity in the heterodimeric functional unit. A fifth group is the receptor tyrosine phosphatases (RTP), which appear to lack cognate ligands, but may be triggered by events such as cell:cell contact and have identified roles in the skeletal, hematopoietic and immune systems.

A sixth group of catalytic receptors in the Guide is the integrins, which have roles in cell:cell communication, often associated with signaling in the blood.

## Family structure

5981 Cytokine receptor family

5981 IL‐2 receptor family

5983 IL‐3 receptor family

5983 IL‐6 receptor family

5985 IL‐12 receptor family

5985 Prolactin receptor family

5986 Interferon receptor family

5987 IL‐10 receptor family

5988 Immunoglobulin‐like family of IL‐1 receptors

5989 IL‐17 receptor family

5990 GDNF receptor family

5991 Integrins

5994 Natriuretic peptide receptor family

5996 Pattern recognition receptors

5996 Toll‐like receptor family

5997 NOD‐like receptor family

Ű Receptor kinases

Ű Other protein kinases

Ű TK: Tyrosine kinase

5999 Receptor serine/threonine kinase (RSTK) family

6000 Type I receptor serine/threonine kinases

6001 Type II receptor serine/threonine kinases

6001 Type III receptor serine/threonine kinases

6002 RSTK functional heteromers

6003 Receptor tyrosine kinases

6004 Type I RTKs: ErbB (epidermal growth factor) receptor family

6005 Type II RTKs: Insulin receptor family

6005 Type III RTKs: PDGFR, CSFR, Kit, FLT3 receptor family

6007 Type IV RTKs: VEGF (vascular endothelial growth factor) receptor family

6008 Type V RTKs: FGF (fibroblast growth factor) receptor family

6008 Type VI RTKs: PTK7/CCK4

6009 Type VII RTKs: Neurotrophin receptor/Trk family

6010 Type VIII RTKs: ROR family

6010 Type IX RTKs: MuSK

6010 Type X RTKs: HGF (hepatocyte growth factor) receptor family

6011 Type XI RTKs: TAM (TYRO3‐, AXL‐ and MER‐TK) receptor family

6012 Type XII RTKs: TIE family of angiopoietin receptors

6012 Type XIII RTKs: Ephrin receptor family

6013 Type XIV RTKs: RET

6014 Type XV RTKs: RYK

6014 Type XVI RTKs: DDR (collagen receptor) family

6015 Type XVII RTKs: ROS receptors

6015 Type XVIII RTKs: LMR family

6016 Type XIX RTKs: Leukocyte tyrosine kinase (LTK) receptor family

6016 Type XX RTKs: STYK1

Ű TKL: Tyrosine kinase‐like

6017 Receptor tyrosine phosphatases (RTP)

6018 Tumour necrosis factor (TNF) receptor family

## Cytokine receptor family

### Overview

Cytokines are not a clearly defined group of agents, other than having an impact on immune signalling pathways, although many cytokines have effects on other systems, such as in development. A feature of some cytokines, which allows them to be distinguished from hormones, is that they may be produced by “non‐secretory” cells, for example, endothelial cells. Within the cytokine receptor family, some subfamilies may be identified, which are described elsewhere in the Guide to PHARMACOLOGY, receptors for the TNF family, the TGF‐*β* family and the chemokines. Within this group of records are described Type I cytokine receptors, typified by interleukin receptors, and Type II cytokine receptors, exemplified by interferon receptors. These receptors possess a conserved extracellular region, known as the cytokine receptor homology domain (CHD), along with a range of other structural modules, including extracellular immunoglobulin (Ig)‐like and fibronectin type III (FBNIII)‐like domains, a transmembrane domain, and intracellular homology domains. An unusual feature of this group of agents is the existence of soluble and decoy receptors. These bind cytokines without allowing signalling to occur. A further attribute is the production of endogenous antagonist molecules, which bind to the receptors selectively and prevent signalling. A commonality of these families of receptors is the ligand‐induced homo‐ or hetero‐oligomerisation, which results in the recruitment of intracellular protein partners to evoke cellular responses, particularly in inflammatory or haematopoietic signalling. Although not an exclusive signalling pathway, a common feature of the majority of cytokine receptors is activation of the JAK/STAT pathway. This cascade is based around the protein tyrosine kinase activity of the Janus kinases (JAK), which phosphorylate the receptor and thereby facilitate the recruitment of signal transducers and activators of transcription (STATs). The activated homo‐ or heterodimeric STATs function principally as transcription factors in the nucleus.

**Type I cytokine receptors** are characterized by two pairs of conserved cysteines linked via disulfide bonds and a C‐terminal WSXWS motif within their CHD. Type I receptors are commonly classified into five groups, based on sequence and structual homology of the receptor and its cytokine ligand, which is potentially more reflective of evolutionary relationships than an earlier scheme based on the use of common signal transducing chains within a receptor complex. These are the IL‐2, IL‐3, IL‐6, IL‐12 and prolactin families.

**Type II cytokine receptors** also have two pairs of conserved cysteines but with a different arrangement to Type I and also lack the WSXWS motif. The type II cytokine receptors include the interferon, IL‐10, IL‐1 and IL‐17 receptors.

## IL‐2 receptor family

### Overview

The IL‐2 receptor family consists of one or more ligand‐selective subunits, and a common *γ* chain (*γ*c): *IL2RG*, P31785), though IL‐4 and IL‐7 receptors can form complexes with other receptor chains. Receptors of this family associate with Jak1 and Jak3, primarily activating Stat5, although certain family members can also activate Stat1, Stat3, or Stat6. Ro264550 has been described as a selective IL‐2 receptor antagonist, which binds to IL‐2 177.

**Table:** 

Nomenclature	Interleukin‐2 receptor	Interleukin‐4 receptor type I	Interleukin‐4 receptor type II	Interleukin‐7 receptor	Interleukin‐9 receptor
Subunits	Interleukin‐2 receptor subunit *β* (Ligand‐binding subunit), Interleukin‐2 receptor subunit *γ* (Other subunit), Interleukin‐2 receptor subunit *α* (Ligand‐binding subunit)	Interleukin‐4 receptor subunit *α* (Ligand‐binding subunit), Interleukin‐2 receptor subunit *γ* (Other subunit)	Interleukin‐13 receptor subunit *α*1 (Other subunit), Interleukin‐4 receptor subunit *α* (Ligand‐binding subunit)	Interleukin‐2 receptor subunit *γ* (Other subunit), Interleukin‐7 receptor subunit *α* (Ligand‐binding subunit)	Interleukin‐2 receptor subunit *γ* (Other subunit), Interleukin 9 receptor (Ligand‐binding subunit)
Endogenous agonists	IL‐2 (*IL2*, P60568)	IL‐4 (*IL4*, P05112)	IL‐13 (*IL13*, P35225), IL‐4 (*IL4*, P05112)	IL‐7 (*IL7*, P13232)	IL‐9 (*IL9*, P15248)
Endogenous antagonists	IL‐1 receptor antagonist (*IL1RN*, P18510)	–	–	–	–
Antagonists	Ro26‐4550 177	–	–	–	–
Selective antagonists	AF12198 [3]	–	–	–	–

**Table:** 

Nomenclature	Interleukin 13 receptor, *α*2	Interleukin‐15 receptor	Interleukin‐21 receptor	Thymic stromal lymphopoietin receptor
HGNC, UniProt	*IL13RA2*, Q14627	–	–	–
Subunits	–	Interleukin‐2 receptor subunit *β* (Ligand‐binding subunit), Interleukin‐15 receptor subunit *α* (Ligand‐binding subunit), Interleukin‐2 receptor subunit *γ* (Other subunit)	Interleukin‐2 receptor subunit *γ* (Other subunit), Interleukin 21 receptor (Ligand‐binding subunit)	Cytokine receptor‐like factor 2 (Other subunit), Interleukin‐7 receptor subunit *α* (Ligand‐binding subunit)
Endogenous agonists	–	IL‐15 (*IL15*, P40933)	IL‐21 (*IL21*, Q9HBE4)	thymic stromal lymphopoietin (*TSLP*, Q969D9)
Comments	Decoy receptor that binds IL‐13 (*IL13*, P35225) as a monomer.	–	–	–

Subunits

**Table:** 

Nomenclature	Interleukin‐2 receptor subunit *α*	Interleukin‐2 receptor subunit *β*	Interleukin‐2 receptor subunit *γ*	Interleukin‐4 receptor subunit *α*	Interleukin‐7 receptor subunit *α*
HGNC, UniProt	*IL2RA*, P01589	*IL2RB*, P14784	*IL2RG*, P31785	*IL4R*, P24394	*IL7R*, P16871
Antibodies	daclizumab (Binding) (p*K*_d_>8) 154, basiliximab (Binding)	–	–	dupilumab (Binding) (pIC_50_ 11.1) 121	–

**Table:** 

Nomenclature	Interleukin 9 receptor	Interleukin‐13 receptor subunit *α*1	Interleukin‐15 receptor subunit *α*	Interleukin 21 receptor	Cytokine receptor‐like factor 2
HGNC, UniProt	*IL9R*, Q01113	*IL13RA1*, P78552	*IL15RA*, Q13261	*IL21R*, Q9HBE5	*CRLF2*, Q9HC73

## IL‐3 receptor family

### Overview

The IL‐3 receptor family signal through a receptor complex comprising of a ligand‐specific *α* subunit and a common *β* chain (*CSF2RB*, P32927), which is associated with Jak2 and signals primarily through Stat5.

**Table:** 

Nomenclature	Interleukin‐3 receptor	Interleukin‐5 receptor	Granulocyte macrophage colony‐stimulating factor receptor
Subunits	Interleukin 3 receptor, *α* subunit (Ligand‐binding subunit), Cytokine receptor common *β* subunit (Other subunit)	Interleukin 5 receptor, *α* subunit (Ligand‐binding subunit), Cytokine receptor common *β* subunit (Other subunit)	GM‐CSF receptor, *α* subunit (Ligand‐binding subunit), Cytokine receptor common *β* subunit (Other subunit)
Endogenous agonists	IL‐3 (*IL3*, P08700)	IL‐5 (*IL5*, P05113)	G‐CSF (*CSF3*, P09919), GM‐CSF (*CSF2*, P04141)
Selective antagonists	–	YM90709 [133]	–

Subunits

**Table:** 

Nomenclature	Interleukin 3 receptor, *α* subunit	Interleukin 5 receptor, *α* subunit	GM‐CSF receptor, *α* subunit	Cytokine receptor common *β* subunit
HGNC, UniProt	*IL3RA*, P26951	*IL5RA*, Q01344	*CSF2RA*, P15509	*CSF2RB*, P32927
Endogenous agonists	IL‐3 (*IL3*, P08700)	IL‐5 (*IL5*, P05113)	GM‐CSF (*CSF2*, P04141)	–
Antibodies	–	benralizumab (Binding) (p*K*_d_ 8.7) 93	mavrilimumab (Binding) (pIC_50_ 9.9) 29	–

## IL‐6 receptor family

### Overview

The IL‐6 receptor family signal through a ternary receptor complex consisting of the cognate receptor and either the IL‐6 signal transducer gp130 (*IL6ST*, P40189) or the oncostatin M‐specific receptor, *β* subunit (*OSMR,* Q99650), which then activates the JAK/STAT, Ras/Raf/MAPK and PI 3‐kinase/PKB signalling modules. Unusually amongst the cytokine receptors, the CNTF receptor is a glycerophosphatidylinositol‐linked protein.

**Table:** 

Nomenclature	Interleukin‐6 receptor	Interleukin‐11 receptor	Interleukin‐31 receptor	Ciliary neutrophic factor receptor
Subunits	Interleukin‐6 receptor, *α* subunit (Ligand‐binding subunit), Interleukin‐6 receptor, *β* subunit (Other subunit)	Interleukin‐11 receptor, *α* subunit (Ligand‐binding subunit), Interleukin‐6 receptor, *β* subunit (Other subunit)	Interleukin‐31 receptor, *α* subunit (Ligand‐binding subunit), Oncostatin M‐specific receptor, *β* subunit (Other subunit)	Leukemia inhibitory factor receptor (Other subunit), Interleukin‐6 receptor, *β* subunit, Ciliary neurotrophic factor receptor *α* subunit (Ligand‐binding subunit)
Endogenous agonists	IL‐6 (*IL6*, P05231)	IL‐11 (*IL11*, P20809)	IL‐31 (*IL31*, Q6EBC2)	CRCF1/CLCF1 heterodimer (*CLCF1* *CRLF1*, O75462 Q9UBD9), ciliary neurotrophic factor (*CNTF*, P26441)
Agonists	–	oprelvekin	–	–
Antibodies	tocilizumab (Binding) (p*K*_d_ 8.6)	–	–	–

**Table:** 

Nomenclature	Leptin receptor	Leukemia inhibitory factor receptor	Oncostatin‐M receptor	Interleukin‐27 receptor
HGNC, UniProt	*LEPR*, P48357	–	–	–
Subunits	–	Leukemia inhibitory factor receptor (Ligand‐binding subunit), Interleukin‐6 receptor, *β* subunit (Other subunit)	Interleukin‐6 receptor, *β* subunit (Other subunit), Oncostatin M‐specific receptor, *β* subunit (Ligand‐binding subunit)	Interleukin‐6 receptor, *β* subunit (Other subunit), Interleukin 27 receptor, alpha (Ligand‐binding subunit)
Endogenous agonists	leptin (*LEP*, P41159)	LIF (*LIF*, P15018), cardiotrophin‐1 (*CTF1*, Q16619), oncostatin M (*OSM*, P13725)	oncostatin M (*OSM*, P13725)	IL‐27 (*EBI3* *IL27*, Q14213 Q8NEV9)

Subunits

**Table:** 

Nomenclature	Interleukin‐6 receptor, *α* subunit	Interleukin‐6 receptor, *β* subunit	Interleukin‐11 receptor, *α* subunit	Interleukin 27 receptor, alpha
HGNC, UniProt	*IL6R*, P08887	*IL6ST*, P40189	*IL11RA*, Q14626	*IL27RA*, Q6UWB1
Antibodies	sarilumab (Binding) (p*K*_d_ 10.6–11.1) 171	–	–	–

**Table:** 

Nomenclature	Interleukin‐31 receptor, *α* subunit	Ciliary neurotrophic factor receptor *α* subunit	Leukemia inhibitory factor receptor	Oncostatin M‐specific receptor, *β* subunit
HGNC, UniProt	*IL31RA*, Q8NI17	*CNTFR*, P26992	*LIFR*, P42702	*OSMR*, Q99650

## IL‐12 receptor family

### Overview

IL‐12 receptors are a subfamily of the IL‐6 receptor family. IL12RB1 is shared between receptors for IL‐12 and IL‐23; the functional agonist at IL‐12 receptors is a heterodimer of IL‐12A/IL‐12B, while that for IL‐23 receptors is a heterodimer of IL‐12B/IL‐23A.

**Table:** 

Nomenclature	Interleukin‐12 receptor	Interleukin‐23 receptor	Interleukin‐12 receptor, *β*1subunit	Interleukin‐12 receptor, *β*2subunit	Interleukin 23 receptor
HGNC, UniProt	–	–	*IL12RB1*, P42701	*IL12RB2*, Q99665	*IL23R*, Q5VWK5
Subunits	Interleukin‐12 receptor, *β*2 subunit (Other subunit), Interleukin‐12 receptor, *β*1 subunit (Ligand‐binding subunit)	Interleukin 23 receptor (Ligand‐binding subunit), Interleukin‐12 receptor, *β*1 subunit (Ligand‐binding subunit)	–	–	–
Endogenous agonists	IL‐12 (*IL12A* *IL12B*, P29459 P29460)	IL‐23 (*IL12B* *IL23A*, P29460)	–	–	–

## Prolactin receptor family

### Overview

Prolactin family receptors form homodimers in the presence of their respective ligands, associate exclusively with Jak2 and signal via Stat5.

**Table:** 

Nomenclature	Eythropoietin receptor	Granulocyte colony‐stimulating factor receptor	Growth hormone receptor	Prolactin receptor	Thrombopoietin receptor
HGNC, UniProt	*EPOR*, P19235	*CSF3R*, Q99062	*GHR*, P10912	*PRLR*, P16471	*MPL*, P40238
Endogenous agonists	erythropoietin (*EPO*, P01588) (Selective) (pIC_50_ 11.1) [48]	G‐CSF (*CSF3*, P09919)	growth hormone 1 (*GH1*, P01241), growth hormone 2 (*GH2*, P01242)	choriomammotropin (*CSH1* *CSH2*, P01243), chorionic somatomammotropinhormone‐like 1 (*CSHL1*, Q14406), prolactin (*PRL*, P01236)	thrombopoietin (*THPO*, P40225)
Agonists	peginesatide (pIC_50_ 10.4) [48]	pegfilgrastim	–	–	romiplostim
Selective agonists	–	–	–	–	eltrombopag (pEC_50_ 7.4) [119]
Antagonists	–	–	pegvisomant [180]	–	–

## Interferon receptor family

### Overview

The interferon receptor family includes receptors for type I (*α*, *β* *κ* and *ω*) and type II (*γ*) interferons. There are at least 13 different genes encoding IFN‐*α* subunits in a cluster on human chromosome 9p22: *α*1 (*IFNA1*, P01562), *α*2 (*IFNA2*, P01563), *α*4 (*IFNA4*, P05014), *α*5 (*IFNA5*, P01569), *α*6 (*IFNA6*, P05013), *α*7 (*IFNA7*, P01567), *α*8 (*IFNA8*, P32881), *α*10 (*IFNA10*, P01566), *α*13 (*IFNA13*, P01562), *α*14 (*IFNA14*, P01570), *α*16 (*IFNA16*, P05015), *α*17 (*IFNA17*, P01571) and *α*21 (*IFNA21*, P01568).

**Table:** 

Nomenclature	Interferon‐*α*/*β* receptor	Interferon‐*γ* receptor
Subunits	Interferon *α*/*β* receptor 2 (Other subunit), interferon *α*/*β* receptor 1 (Ligand‐binding subunit)	Interferon *γ* receptor 2 (Other subunit), Interferon *γ* receptor 1 (Ligand‐binding subunit)
Endogenous agonists	IFN‐*α*1/13 (*IFNA1* *IFNA13*, P01562), IFN‐*α*10 (*IFNA10*, P01566), IFN‐*α*14 (*IFNA14*, P01570), IFN‐*α*16 (*IFNA16*, P05015), IFN‐*α*17 (*IFNA17*, P01571), IFN‐*α*2 (*IFNA2*, P01563), IFN‐*α*21 (*IFNA21*, P01568), IFN‐*α*4 (*IFNA4*, P05014), IFN‐*α*5 (*IFNA5*, P01569), IFN‐*α*6 (*IFNA6*, P05013), IFN‐*α*7 (*IFNA7*, P01567), IFN‐*α*8 (*IFNA8*, P32881), IFN‐*β* (*IFNB1*, P01574), IFN‐*κ* (*IFNK*, Q9P0W0), IFN‐*ω* (*IFNW1*, P05000)	IFN‐*γ* (*IFNG*, P01579)
Selective agonists	peginterferon alfa‐2b [191]	–

Subunits

**Table:** 

Nomenclature	interferon *α*/*β* receptor 1	Interferon *α*/*β* receptor 2	Interferon *γ* receptor 1	Interferon *γ* receptor 2
HGNC, UniProt	*IFNAR1*, P17181	*IFNAR2*, P48551	*IFNGR1*, P15260	*IFNGR2*, P38484
Selective agonists	peginterferon alfa‐2b [191]	–	–	–
Antibodies	anifrolumab (Binding) (p*K*_d_>10) 21	–	–	–

## IL‐10 receptor family

### Overview

The IL‐10 family of receptors are heterodimeric combinations of family members: IL10RA/IL10RB responds to IL‐10; IL20RA/IL20RB responds to IL‐19, IL‐20 and IL‐24; IL22RA1/IL20RB responds to IL‐20 and IL‐24; IL22RA1/IL10RB responds to IL‐22; IL28RA/IL10RB responds to IL‐28A, IL28B and IL‐29.

**Table:** 

Nomenclature	Interleukin‐10 receptor	Interleukin‐20 receptor	Interleukin‐22*α*1/20*β* heteromer	Interleukin‐22*α*1/10*β* heteromer	Interleukin‐22 receptor *α*2	Interferon‐*λ* receptor 1
HGNC, UniProt	–	–	–	–	*IL22RA2*, Q969J5	–
Subunits	Interleukin 10 receptor,*α* subunit (Ligand‐binding subunit), Interleukin 10 receptor,*β* subunit (Other subunit)	Interleukin 20 receptor, *β* subunit (Other subunit), Interleukin 20 receptor, *α* subunit (Ligand‐binding subunit)	Interleukin 22 receptor,*α*1 subunit (Ligand‐binding subunit), Interleukin 20 receptor,*β* subunit (Ligand‐binding subunit)	Interleukin 22 receptor,*α*1 subunit (Ligand‐binding subunit), Interleukin 10 receptor,*β* subunit (Ligand‐binding subunit)	–	Interferon‐*λ* receptor subunit 1 (Ligand‐binding subunit), Interleukin 10 receptor, *β* subunit (Other subunit)
Endogenous agonists	IL‐10 (*IL10*, P22301)	IL‐19 (*IL19*, Q9UHD0), IL‐20 (*IL20*, Q9NYY1), IL‐24 (*IL24*, Q13007)	IL‐20 (*IL20*, Q9NYY1), IL‐24 (*IL24*, Q13007)	IL‐22 (*IL22*, Q9GZX6)	–	IFN‐*λ*1 (*IFNL1*, Q8IU54), IFN‐*λ*2 (*IFNL2*, Q8IZJ0), IFN‐*λ*3 (*IFNL3*, Q8IZI9)
Comments	–	–	–	–	Soluble decoy receptor that binds IL‐22 (*IL22*, Q9GZX6) as a monomer.	–

Subunits

**Table:** 

Nomenclature	Interleukin 10 receptor,*α* subunit	Interleukin 10 receptor,*β* subunit	Interleukin 20 receptor,*α* subunit	Interleukin 20 receptor,*β* subunit	Interleukin 22 receptor,*α*1 subunit	Interferon‐*λ*receptor subunit 1
HGNC, UniProt	*IL10RA*, Q13651	*IL10RB*, Q08334	*IL20RA*, Q9UHF4	*IL20RB*, Q6UXL0	*IL22RA1*, Q8N6P7	*IFNLR1*, Q8IU57

## Immunoglobulin‐like family of IL‐1 receptors

### Overview

The immunoglobulin‐like family of IL‐1 receptors are heterodimeric receptors made up of a cognate receptor subunit and an IL‐1 receptor accessory protein, *IL1RAP* (Q9NPH3, also known as C3orf13, IL‐1RAcP, IL1R3). They are characterised by extracellular immunoglobulin‐like domains and an intracellular Toll/Interleukin‐1R (TIR) domain.

**Table:** 

Nomenclature	Interleukin‐1 receptor, type I	Interleukin‐33 receptor	Interleukin‐36 receptor	Interleukin‐1 receptor, type II	Interleukin‐18 receptor
Subunits	IL‐1 receptor accessory protein (Other subunit), Interleukin 1 receptor, type I (Ligand‐binding subunit)	IL‐1 receptor accessory protein (Other subunit), Interleukin‐1 receptor‐like 1 (Ligand‐binding subunit)	IL‐1 receptor accessory protein (Other subunit), Interleukin‐1 receptor‐like 2 (Ligand‐binding subunit)	IL‐1 receptor accessory protein (Other subunit), Interleukin 1 receptor, type II (Ligand‐binding subunit)	IL‐18 receptor accessory protein (Other subunit), Interleukin‐18 1 (Ligand‐binding subunit)
Inhibitors	anakinra (p*K*_d_ 7.8) [44]	–	–	–	–
Endogenous agonists	IL‐1*α* (*IL1A*, P01583), IL‐1*β* (*IL1B*, P01584)	IL‐33 (*IL33*, O95760)	IL‐36*α* (*IL36A*, Q9UHA7), IL‐36*β* (*IL36B*, Q9NZH7), IL‐36*γ* (*IL36G*, Q9NZH8)	–	IL‐18 (*IL18*, Q14116), IL‐37 (*IL37*, Q9NZH6)
Endogenous antagonists	IL‐1 receptor antagonist (*IL1RN*, P18510)	–	IL‐36 receptor antagonist (*IL36RN*, Q9UBH0)	–	–
Selective antagonists	AF12198 [3]	–	–	–	–
Comments	–	–	IL‐36 receptor antagonist (*IL36RN*, Q9UBH0) is a highly specific antagonist of the response to IL‐36*γ* (*IL36G*, Q9NZH8).	Decoy receptor that binds IL‐1*α* (*IL1A*, P01583), IL‐1*β* (*IL1B*, P01584) and IL‐1 receptor antagonist (*IL1RN*, P18510).	–

Subunits

**Table:** 

Nomenclature	Interleukin 1 receptor, type I	Interleukin 1 receptor, type II	Interleukin‐1 receptor‐like 1	Interleukin‐1 receptor‐like 2	Interleukin‐18 1
HGNC, UniProt	*IL1R1*, P14778	*IL1R2*, P27930	*IL1RL1*, Q01638	*IL1RL2*, Q9HB29	*IL18R1*, Q13478

## IL‐17 receptor family

### Overview

The IL17 cytokine family consists of six ligands (IL‐17A‐F), which signal through five receptors (IL‐17RA‐E).

**Table:** 

Nomenclature	Interleukin‐17 receptor	Interleukin‐25 receptor	Interleukin‐17C receptor
Subunits	Interleukin 17 receptor A (Ligand‐binding subunit), interleukin 17 receptor C (Other subunit)	Interleukin 17 receptor B (Ligand‐binding subunit), Interleukin 17 receptor A (Other subunit)	Interleukin 17 receptor A (Other subunit), Interleukin 17 receptor E (Ligand‐binding subunit)
Endogenous agonists	IL‐17A (*IL17A*, Q16552), IL‐17A/IL‐17F (*IL17A* *IL17F*, Q16552 Q96PD4), IL‐17F (*IL17F*, Q96PD4)	IL‐17B (*IL17B*, Q9UHF5), IL‐25 (*IL25*, Q9H293)	IL‐17C (*IL17C*, Q9P0M4)

Subunits

**Table:** 

Nomenclature	Interleukin 17 receptor A	Interleukin 17 receptor B	interleukin 17 receptor C	Interleukin‐17 receptor D	Interleukin 17 receptor E
HGNC, UniProt	*IL17RA*, Q96F46	*IL17RB*, Q9NRM6	*IL17RC*, Q8NAC3	*IL17RD*, Q8NFM7	*IL17RE*, Q8NFR9
Antibodies	brodalumab (Binding) (p*K*_d_ 9.2) 179	–	–	–	–
Comments	–	–	–	The endogenous agonist for this receptor is unknown.	–

### Further Reading

Broughton SE *et al*. (2012) The GM‐CSF/IL‐3/IL‐5 cytokine receptor family: from ligand recognition to initiation of signaling. *Immunol. Rev.* **250**: 277‐302 [PMID:23046136]

Chang SH *et al*. (2011) Signaling of interleukin‐17 family cytokines in immunity and inflammation. *Cell. Signal.* **23**: 1069‐75 [PMID:21130872]

George PM *et al*. (2012) Pharmacology and therapeutic potential of interferons. *Pharmacol. Ther.* **135**: 44‐53 [PMID:22484806]

Gibbert K *et al*. (2013) IFN‐*α* subtypes: distinct biological activities in anti‐viral therapy. *Br. J. Pharmacol.* **168**: 1048‐58 [PMID:23072338]

Mackall CL *et al*. (2011) Harnessing the biology of IL‐7 for therapeutic application. *Nat. Rev. Immunol.* **11**: 330‐42 [PMID:21508983]

Mihara M *et al*. (2012) IL‐6/IL‐6 receptor system and its role in physiological and pathological conditions. *Clin. Sci.* **122**: 143‐59 [PMID:22029668]

Miossec P *et al*. (2012) Targeting IL‐17 and TH17 cells in chronic inflammation. *Nat Rev Drug Discov* **11**: 763‐76 [PMID:23023676]

Murugaiyan G *et al*. (2013) IL‐27 in tumor immunity and immunotherapy. *Trends Mol Med* **19**: 108‐16 [PMID:23306374]

Palmer G *et al*. (2011) Interleukin‐33 biology with potential insights into human diseases. *Nat Rev Rheumatol* **7**: 321‐9 [PMID:21519352]

Pappu R *et al*. (2011) The interleukin‐17 cytokine family: critical players in host defence and inflammatory diseases. *Immunology* **134**: 8‐16 [PMID:21726218]

Rincon M. (2012) Interleukin‐6: from an inflammatory marker to a target for inflammatory diseases. *Trends Immunol.* **33**: 571‐7 [PMID:22883707]

Rubino SJ *et al*. (2012) Innate IL‐17 and IL‐22 responses to enteric bacterial pathogens. *Trends Immunol.* **33**: 112‐8 [PMID:22342740]

Tanaka T *et al*. (2012) Therapeutic targeting of the interleukin‐6 receptor. *Annu. Rev. Pharmacol. Toxicol.* **52**: 199‐219 [PMID:21910626]

Wojno ED *et al*. (2012) New directions in the basic and translational biology of interleukin‐27. *Trends Immunol.* **33**: 91‐7 [PMID:22177689]

Zhu S *et al*. (2012) IL‐17/IL‐17 receptor system in autoimmune disease: mechanisms and therapeutic potential. *Clin. Sci.* **122**: 487‐511 [PMID:22324470]

## GDNF receptor family

### Overview

GDNF family receptors (provisional nomenclature) are extrinsic tyrosine kinase receptors. Ligand binding to the extracellular domain of the glycosylphosphatidylinositol‐linked cell‐surface receptors (tabulated below) activates a transmembrane tyrosine kinase enzyme, RET (see Receptor Tyrosine Kinases). The endogenous ligands are typically dimeric, linked through disulphide bridges: glial cell‐derived neurotrophic factor GDNF (*GDNF*, P39905) (211 aa); neurturin (*NRTN*, Q99748) (197 aa); artemin (*ARTN*, Q5T4W7) (237 aa) and persephin (*PSPN*, O60542) (PSPN, 156 aa).

**Table:** 

Nomenclature	GDNF family receptor *α*1	GDNF family receptor *α*2	GDNF family receptor *α*3	GDNF family receptor *α*4
Common abreviation	GFR*α*1	GFR*α*2	GFR*α*3	GFR*α*4
HGNC, UniProt	*GFRA1*, P56159	*GFRA2*, O00451	*GFRA3*, O60609	*GFRA4*, Q9GZZ7
Potency order	GDNF (*GDNF*, P39905) >neurturin (*NRTN*, Q99748) >artemin (*ARTN*, Q5T4W7)	neurturin (*NRTN*, Q99748) >GDNF (*GDNF*, P39905)	artemin (*ARTN*, Q5T4W7)	persephin (*PSPN*, O60542)
Labelled ligands	[^125^I]GDNF (rat) (p*K*_d_ 10.2–11.5) [92, 182]	–	–	–

### Comments

Inhibitors of other receptor tyrosine kinases, such as semaxanib, which inhibits VEGF receptor function, may also inhibit Ret function [131]. Mutations of RET and GDNF genes may be involved in Hirschsprung's disease, which is characterized by the absence of intramural ganglion cells in the hindgut, often resulting in intestinal obstruction.

### Further Reading

Allen SJ *et al*. (2013) GDNF, NGF and BDNF as therapeutic options for neurodegeneration. *Pharmacol. Ther.* **138**: 155‐75 [PMID:23348013]

Carnicella S *et al*. (2009) GDNF–a potential target to treat addiction. *Pharmacol. Ther.* **122**: 9‐18 [PMID:19136027]

Liu H *et al*. (2012) Role of glial cell line‐derived neurotrophic factor in perineural invasion of pancreatic cancer. *Biochim. Biophys. Acta* **1826**: 112‐20 [PMID:22503821]

Mickiewicz AL *et al*. (2011) GDNF family ligands: a potential future for Parkinson's disease therapy. *CNS Neurol Disord Drug Targets* **10**: 703‐11 [PMID:21838676]

Pascual A *et al*. (2011) GDNF and protection of adult central catecholaminergic neurons. *J. Mol. Endocrinol.* **46**: R83‐92 [PMID:21357726]

Rangasamy SB *et al*. (2010) Neurotrophic factor therapy for Parkinson's disease. *Prog. Brain Res.* **184**: 237‐64 [PMID:20887879]

## Integrins

### Overview

Integrins are unusual signalling proteins that function to signal both from the extracellular environment into the cell, but also from the cytoplasm to the external of the cell. The intracellular signalling cascades associated with integrin activation focus on protein kinase activities, such as focal adhesion kinase and Src. Based on this association between extracellular signals and intracellular protein kinase activity, we have chosen to include integrins in the ‘Catalytic receptors’ section of the database until more stringent criteria from NC‐IUPHAR allows precise definition of their classification.

Integrins are heterodimeric entities, composed of *α* and *β* subunits, each 1TM proteins, which bind components of the extracellular matrix or counter‐receptors expressed on other cells. One class of integrin contains an inserted domain (I) in its *α* subunit, and if present (in *α*1, *α*2, *α*10, *α*11, *α*D, *α*E, *α*L, *α*M and *α*X), this I domain contains the ligand binding site. All *β* subunits possess a similar I‐like domain, which has the capacity to bind ligand, often recognising the RGD motif. The presence of an *α* subunit I domain precludes ligand binding through the *β* subunit. Integrins provide a link between ligand and the actin cytoskeleton (through typically short intracellular domains). Integrins bind several divalent cations, including a Mg^2+^ ion in the I or I‐like domain that is essential for ligand binding. Other cation binding sites may regulate integrin activity or stabilise the 3D structure. Integrins regulate the activity of particular protein kinases, including focal adhesion kinase and integrin‐linked kinase. Cellular activation regulates integrin ligand affinity via inside‐out signalling and ligand binding to integrins can regulate cellular activity via outside‐in signalling.

**Table:** 

Nomenclature	integrin *α*1*β*1	integrin *α*2*β*1	integrin *α*IIb*β*3	integrin *α*4*β*1
Subunits	integrin, beta 1 subunit(fibronectin receptor, beta polypeptide,antigen CD29 includes MDF2, MSK12), integrin, alpha 1 subunit	integrin, beta 1 subunit(fibronectin receptor, beta polypeptide,antigen CD29 includes MDF2, MSK12), integrin, alpha 2 subunit(CD49B, alpha 2 subunitof VLA‐2 receptor)	integrin, beta 3 subunit (platelet glycoprotein IIIa, antigen CD61), integrin, alpha IIb subunit (platelet glycoprotein IIb of IIb/IIIa complex, antigen CD41)	integrin, beta 1 subunit (fibronectin receptor, beta polypeptide, antigen CD29 includes MDF2, MSK12), integrin, alpha 4 subunit (antigen CD49D, alpha 4 subunit of VLA‐4 receptor)
Ligands	collagen, laminin	collagen, laminin, thrombospondin	fibrinogen (*FGA* *FGB* *FGG*, P02671 P02675 P02679), fibronectin (*FN1*, P02751), von Willebrand factor (*VWF*, P04275), vitronectin (*VTN*, P04004), thrombospondin	fibronectin (*FN1*, P02751), vascular cell adhesion protein 1 (*VCAM1*, P19320), osteopontin (*SPP1*, P10451), thrombospondin
Inhibitors	obtustatin (pIC_50_ 9.1) [118]	TCI15 (pIC_50_ 7.9) [128]	G4120 [124], GR 144053, eptifibatide, tirofiban	BIO1211 (pIC_50_ 8.3–9) [108], TCS2314
Antibodies	–	–	abciximab (Binding) 31	natalizumab (Inhibition) [1]
Comments	–	–	–	LDV‐FITC is used as a probe at this receptor.

**Table:** 

Nomenclature	integrin *α*4*β*7	integrin *α*5*β*1	integrin *α*6*β*1	integrin *α*10*β*1
Subunits	integrin, alpha 4 subunit (antigen CD49D, alpha 4 subunit of VLA‐4 receptor), integrin, beta 7 subunit	integrin, beta 1 subunit(fibronectin receptor, beta polypeptide, antigen CD29includes MDF2, MSK12), integrin, alpha 5 subunit(fibronectin receptor, alphapolypeptide)	integrin, beta 1 subunit(fibronectin receptor, betapolypeptide, antigen CD29 includesMDF2, MSK12), integrin, alpha 6 subunit	integrin, beta 1 subunit(fibronectin receptor, betapolypeptide, antigen CD29includes MDF2, MSK12), integrin, alpha 10 subunit
Ligands	–	fibronectin (*FN1*, P02751)	laminin	collagen
Antibodies	vedolizumab (Antagonist) (pIC_50_ 8.3) 151	–	–	–

**Table:** 

Nomenclature	integrin *α*11*β*1	integrin *α*E*β*7	integrin *α*L*β*2	integrin *α*V*β*3
Subunits	integrin, beta 1 subunit(fibronectin receptor, beta polypeptide, antigen CD29includes MDF2, MSK12), integrin, alpha 11 subunit	integrin, alpha E subunit(antigen CD103, human mucosal lymphocyteantigen 1; alpha polypeptide), integrin, beta 7 subunit	integrin, beta 2 subunit (complement component 3 receptor 3 and 4 subunit), integrin, alpha L subunit (antigen CD11A (p180), lymphocyte function‐associated antigen 1; alpha polypeptide)	integrin, beta 3 subunit (platelet glycoprotein IIIa, antigen CD61), integrin, alpha V subunit
Ligands	collagen	E‐cadherin	ICAM‐1 (*ICAM1*, P05362), ICAM‐2 (*ICAM2*, P13598)	vitronectin (*VTN*, P04004), fibronectin (*FN1*, P02751), fibrinogen (*FGA* *FGB* *FGG*, P02671 P02675 P02679), osteopontin (*SPP1*, P10451), von Willebrand factor (*VWF*, P04275), thrombospondin, tenascin
Inhibitors	–	–	A286982 (pIC_50_ 7.4–7.5) [110]	echistatin (pIC_50_ 11.7) [101], P11 (pIC_50_ 11.6) [101], cilengitide (pIC_50_ 8.5) [61]
Antibodies	–	–	–	etaracizumab (Binding) (p*K*_d_ 6.3) 201

### Comments: Integrin ligands

**Collagen** is the most abundant protein in metazoa, rich in glycine and proline residues, made up of cross‐linked triple helical structures, generated primarily by fibroblasts. Extensive post‐translational processing is conducted by prolyl and lysyl hydroxylases, as well as transglutaminases. Over 40 genes for collagen‐*α* subunits have been identified in the human genome. The collagen‐binding integrins *α*1*β*1, *α*2*β*1, *α*10*β*1 and *α*11*β*1 recognise a range of triple‐helical peptide motifs including GFOGER (O = hydroxyproline), a synthetic peptide derived from the primary sequence of collagen I (COL1A1 (*COL1A1*, P02452)) and collagen II (COL2A1 (*COL2A1*, P02458)).

**Laminin** is an extracellular glycoprotein composed of *α*, *β* and *γ* chains, for which five, four and three genes, respectively, are identified in the human genome. It binds to *α*1*β*1, *α*2*β*1, *α*3,*β*1, *α*7*β*1 and *α*6*β*4 integrins10.

**fibrinogen** (***FGA***  ***FGB***  ***FGG***, P02671  **P02675**  **P02679**) is a glycosylated hexamer composed of two *α* (*FGA*, P02671), two *β* (*FGB*, P02675) and two *γ* (*FGG*, P02679,) subunits, linked by disulphide bridges. It is found in plasma and alpha granules of platelets. It forms cross‐links between activated platelets mediating aggregation by binding *α*IIb*β*3; proteolysis by thrombin cleaves short peptides termed fibrinopeptides to generate fibrin, which polymerises as part of the blood coagulation cascade.

**fibronectin** (***FN1***, **P02751**) is a disulphide‐linked homodimer found as two major forms; a soluble dimeric form found in the plasma and a tissue version that is polymeric, which is secreted into the extracellular matrix by fibroblasts. Splice variation of the gene product (*FN1*, P02751) generates multiple isoforms.

**vitronectin** (***VTN***, **P04004**) is a serum glycoprotein and extracellular matrix protein which is found either as a monomer or, following proteolysis, a disulphide ‐linked dimer.

**osteopontin** (***SPP1***, **P10451**) forms an integral part of the mineralized matrix in bone, where it undergoes extensive post‐translation processing, including proteolysis and phosphorylation.

**von Willebrand factor** (***VWF***, **P04275**) is a glycoprotein synthesised in vascular endothelial cells as a disulphide‐linked homodimer, but multimerises further in plasma and is deposited on vessel wall collagen as a high molecular weight multimer. It is responsible for capturing platelets under arterial shear flow (*via* GPIb) and in thrombus propagation (via integrin *α*IIb*β*3).

### Subunits

**Table:** 

Nomenclature	integrin, alpha 1 subunit	integrin, alpha 2 subunit (CD49B, alpha 2 subunit of VLA‐2 receptor)	integrin, alpha IIb subunit (platelet glycoprotein IIbof IIb/IIIa complex,antigen CD41)	integrin, alpha 3 subunit(antigen CD49C, alpha 3 subunit ofVLA‐3 receptor)	integrin, alpha 4 subunit (antigen CD49D, alpha 4 subunit of VLA‐4 receptor)	integrin, alpha 5 subunit (fibronectin receptor, alpha polypeptide)
HGNC, UniProt	*ITGA1*, P56199	*ITGA2*, P08514	*ITGA2B*, P17301	*ITGA3*, P26006	*ITGA4*, P13612	*ITGA5*, P08648
Antibodies	–	–	–	–	natalizumab (Inhibition) [1]	–

**Table:** 

Nomenclature	integrin, alpha 6 subunit	integrin, alpha 7 subunit	integrin, alpha 8 subunit	integrin, alpha 9 subunit	integrin, alpha 10 subunit	integrin, alpha 11 subunit	integrin, alpha D subunit
HGNC, UniProt	*ITGA6*, P23229	*ITGA7*, Q13683	*ITGA8*, P53708	*ITGA9*, Q13797	*ITGA10*, O75578	*ITGA11*, Q9UKX5	*ITGAD*, Q13349

**Table:** 

Nomenclature	integrin, alpha E subunit(antigen CD103, human mucosal lymphocyteantigen 1; alpha polypeptide)	integrin, alpha L subunit (antigen CD11A (p180), lymphocyte function‐associated antigen 1; alpha polypeptide)	integrin, alpha M subunit(complement component 3 receptor 3 subunit)	integrin, alpha V subunit	integrin, alpha X subunit (complement component 3receptor 4 subunit)	integrin, beta 1 subunit (fibronectin receptor, beta polypeptide, antigenCD29 includes MDF2, MSK12)
HGNC, UniProt	*ITGAE*, P38570	*ITGAL*, P20701	*ITGAM*, P11215	*ITGAV*, P06756	*ITGAX*, P20702	*ITGB1*, P05556
Antibodies	–	efalizumab (Binding) (p*K*_d_ 11.4) 81	–	–	–	–

**Table:** 

Nomenclature	integrin, beta 2 subunit(complement component 3receptor 3 and 4 subunit)	integrin, beta 3 subunit(platelet glycoprotein IIIa,antigen CD61)	integrin, beta 4 subunit	integrin, beta 5 subunit	integrin, beta 6 subunit	integrin, beta 7 subunit	integrin, beta 8 subunit
HGNC, UniProt	*ITGB2*, P05107	*ITGB3*, P05106	*ITGB4*, P16144	*ITGB5*, P18084	*ITGB6*, P18564	*ITGB7*, P26010	*ITGB8*, P26012

### Further Reading

Anthis NJ *et al*. (2011) The tail of integrin activation. *Trends Biochem. Sci.* **36**: 191‐8 [PMID:21216149]

Hogg N *et al*. (2011) The insider's guide to leukocyte integrin signalling and function. *Nat. Rev. Immunol.* **11**: 416‐26 [PMID:21597477]

Hynes RO. (2002) Integrins: bidirectional, allosteric signaling machines. *Cell* **110**: 673‐87 [PMID:12297042]

Ivaska J *et al*. (2011) Cooperation between integrins and growth factor receptors in signaling and endocytosis. *Annu. Rev. Cell Dev. Biol.* **27**: 291‐320 [PMID:21663443]

Shattil SJ *et al*. (2010) The final steps of integrin activation: the end game. *Nat. Rev. Mol. Cell Biol.* **11**: 288‐300 [PMID:20308986]

Wickström SA *et al*. (2011) Regulation of membrane traffic by integrin signaling. *Trends Cell Biol.* **21**: 266‐73 [PMID:21440440]

## Natriuretic peptide receptor family

### Overview

Natriuretic peptide receptors (provisional nomenclature) are a family of homodimeric, catalytic receptors with a single TM domain and guanylyl cyclase (EC 4.6.1.2) activity on the intracellular domain of the protein sequence. Isoforms are activated by the peptide hormones atrial natriuretic peptide (*NPPA*, P01160), brain natriuretic peptide (*NPPB*, P16860) and C‐type natriuretic peptide (*NPPC*, P23582). Another family member is GC‐C, the receptor for guanylin (*GUCA2A*, Q02747) and uroguanylin (*GUCA2B*, Q16661) . Family members have conserved ligand‐binding, catalytic (guanylyl cyclase) and regulatory domains with the exception of NPR‐C which has an extracellular binding domain homologous to that of other NPRs, but with a truncated intracellular domain which appears to couple, via the G_i/o_ family of G‐proteins, to activation of phospholipase C, inwardly‐rectifying potassium channels and inhibition of adenylyl cyclase activity [136].

**Table:** 

Nomenclature	guanylate cyclase 2C	NPR‐A	NPR‐B	NPR‐C
HGNC, UniProt	*GUCY2C*, P25092	*NPR1*, P16066	*NPR2*, P20594	*NPR3*, P17342
Potency order	uroguanylin (*GUCA2B*, Q16661) >guanylin (*GUCA2A*, Q02747)	atrial natriuretic peptide (*NPPA*, P01160) ≥brain natriuretic peptide (*NPPB*, P16860) ≫C‐type natriuretic peptide (*NPPC*, P23582) [173]	C‐type natriuretic peptide (*NPPC*, P23582) ≫atrial natriuretic peptide (*NPPA*, P01160) ≫brain natriuretic peptide (*NPPB*, P16860) [173]	atrial natriuretic peptide (*NPPA*, P01160) >C‐type natriuretic peptide (*NPPC*, P23582) ≥brain natriuretic peptide (*NPPB*, P16860) [173]
Endogenous agonists	–	atrial natriuretic peptide (*NPPA*, P01160) (Selective) [144], brain natriuretic peptide (*NPPB*, P16860) (Selective) [144]	C‐type natriuretic peptide (*NPPC*, P23582) (Selective) [173]	osteocrin (*OSTN*, P61366) (Selective) [129]
Selective agonists	linaclotide (p*K*_i_ 8.9) [20, 67], *E. coli* heat‐stable enterotoxin (ST_a_) (p*K*_i_ 8.8) [20]	sANP [144]	–	cANF^4‐23^ [114]
Selective antagonists	–	A‐71915 (p*K*_i_ 9.2–9.5) [41], [Asu7,23']*β*‐ANP‐(7‐28) (p*K*_i_ 7.5) [83], anantin [202]	[Ser^11^](N‐CNP,C‐ANP)pBNP^2‐15^ [42]	AP811 (p*K*_i_ 9.3) [185], M372049 [75]
Labelled ligands	[^125^I]St_a_ (Agonist) (p*K*_d_ 7.8) [66]	[^125^I]ANP (human) (Agonist)	[^125^I]CNP (human)	[^125^I]ANP (human)

### Comments

The polysaccharide obtained from fermentation of Aureobasidium species, HS142‐1, acts as an antagonist at both NPR‐A and NPR‐B receptors [132].

*GUCY2D* (RetGC1, GC‐E, Q02846) and *GUCY2F* (RetGC2, GC‐F, P51841) are predominantly retinal guanylyl cyclase activities, which are inhibited by calcium ions acting through the guanylyl cyclase activating peptides GCAP1 (*GUCA1A*, 43080), GCAP2 (*GUCA1B*, Q9UMX6) and GCAP3 (*GUCA1C*, O95843) [78].

### Further Reading

Kuhn M. (2012) Endothelial actions of atrial and B‐type natriuretic peptides. *Br. J. Pharmacol.* **166**: 522‐31 [PMID:22220582]

Misono KS *et al*. (2011) Structure, signaling mechanism and regulation of the natriuretic peptide receptor guanylate cyclase. *FEBS J.* **278**: 1818‐29 [PMID:21375693]

Pandey KN. (2011) The functional genomics of guanylyl cyclase/natriuretic peptide receptor‐A: perspectives and paradigms. *FEBS J.* **278**: 1792‐807 [PMID:21375691]

Potter LR. (2011) Natriuretic peptide metabolism, clearance and degradation. *FEBS J.* **278**: 1808‐17 [PMID:21375692]

Potter LR. (2011) Regulation and therapeutic targeting of peptide‐activated receptor guanylyl cyclases. *Pharmacol. Ther.* **130**: 71‐82 [PMID:21185863]

Potter LR. (2011) Guanylyl cyclase structure, function and regulation. *Cell. Signal.* **23**: 1921‐6 [PMID:21914472]

## Pattern recognition receptors

### Overview

Pattern Recognition Receptors (PRRs, [174]) (**nomenclature as agreed by NC‐IUPHAR sub‐committee on Pattern Recognition Receptors,** [18]) participate in the innate immune response to microbial agents, the stimulation of which leads to activation of intracellular enzymes and regulation of gene transcription. PRRs include both cell‐surface and intracellular proteins, including toll‐like receptors (TLRs), nucleotide‐binding oligomerization domain‐like receptors (NLRs, also known as NOD‐like receptors) and the mannose receptor family (ENSFM00250000004089). PRRs may be divided into signalling‐associated members, identified here, and endocytic members (such as the mannose receptor family), the function of which appears to be to recognise particular microbial motifs for subsequent cell attachment, internalisation and destruction.

PRRs express multiple leucine‐rich regions to bind a range of microbially‐derived ligands, termed PAMPs or pathogen‐associated molecular patterns, which includes peptides, carbohydrates, peptidoglycans, lipoproteins, lipopolysaccharides, and nucleic acids.

### Further Reading

Bryant CE *et al*. (2015) International Union of Basic and Clinical Pharmacology. XCVI. Pattern recognition receptors in health and disease. *Pharmacol. Rev.* **67**: 462‐504 [PMID:25829385]

Davis BK *et al*. (2011) The inflammasome NLRs in immunity, inflammation, and associated diseases. *Annu. Rev. Immunol.* **29**: 707‐35 [PMID:21219188]

Ting JP *et al*. (2008) The NLR gene family: a standard nomenclature. *Immunity* **28**: 285‐7 [PMID:18341998]

## Toll‐like receptor family

### Overview

Members of the toll‐like family of receptors (nomenclature recommended by the NC‐IUPHAR subcommittee on pattern recognition receptors, [18]) share significant homology with the interleukin‐1 receptor family and appear to require dimerization either as homo‐ or heterodimers for functional activity. Heterodimerization appears to influence the potency of ligand binding substantially (*e.g.* TLR1/2 and TLR2/6, [175, 176]). TLR1, TLR2, TLR4, TLR5, TLR6 and TLR11 are cell‐surface proteins, while other members are associated with intracellular organelles, signalling through the MyD88‐dependent pathways (with the exception of TLR3). As well as responding to exogenous infectious agents, it has been suggested that selected members of the family may be activated by endogenous ligands, such as hsp60 (*HSPD1*, P10809) [141].

**Table:** 

Nomenclature	TLR1	TLR2	TLR3	TLR4	TLR5
HGNC, UniProt	*TLR1*, Q15399	*TLR2*, O60603	*TLR3*, O15455	*TLR4*, O00206	*TLR5*, O60602
Agonists	–	peptidoglycan [165, 205]	polyIC [6]	LPS [150], paclitaxel [85]	flagellin [69]
Comments	Functions as a heterodimer with TLR2 in detection of triacylated lipoproteins. Activated by the synthetic analogue Pam3CSK4.	Functions as a heterodimer with either TLR1 or TLR6 in the detection of triacylated and diacylated lipopeptides respectively. TLR1/2 and 2/6 heterodimers can be activated by the synthetic lipopeptides Pam3CSK4 and Pam2CSK4 respectively. There is some debate in the field as to whether or not peptidoglycan is a direct agonist of TLR2, or whether the early studies reporting this contained contaminating lipoproteins.	Involved in endosomal detection of dsRNA; pro‐inflammatory.	eritoran (E5564) is a lipid A analogue, which has been described as a TLR4 antagonist [80]. TLR4 signals in conjunction with the co‐factor MD2.	Involved in the detection of bacterial flagellin; pro‐inflammatory.

**Table:** 

Nomenclature	TLR6	TLR7	TLR8	TLR9	TLR10	TLR11
HGNC, UniProt	*TLR6*, Q9Y2C9	*TLR7*, Q9NYK1	*TLR8*, Q9NR97	*TLR9*, Q9NR96	*TLR10*, Q9BXR5	–
Agonists	–	imiquimod [72], loxoribine [70], resiquimod [72]	imiquimod, resiquimod [72]	–	–	–
Antagonists	–	hydroxychloroquine (pIC_50_ 5.6) [98]	–	hydroxychloroquine (pIC_50_ 7.1) [98]	–	–
Comments	Functions as a heterodimer with TLR2. Involved in the pro‐inflammatory response to diacylated bacterial lipopeptides.	Activated by imidazoquinoline derivatives and RNA oligoribonucleotides. Involved in endosomal detection of ssRNA; pro‐inflammatory.	Activated by imidazoquinoline derivatives and RNA oligoribonucleotides. Endosomal detection of ssRNA; pro‐inflammatory.	Toll‐like receptor 9 interacts with unmethylated CpG dinucleotides from bacterial DNA [73]. Activated by CpG rich DNA sequences; pro‐inflammatory.	Murine TLR10 has a retroviral insertion that makes in non‐functional.	Found in mouse

### Further Reading

Bryant CE *et al*. (2015) International Union of Basic and Clinical Pharmacology. XCVI. Pattern recognition receptors in health and disease. *Pharmacol. Rev.* **67**: 462‐504 [PMID:25829385]

## NOD‐like receptor family

### Overview

The nucleotide‐binding oligomerization domain, leucine‐rich repeat (NLR) family of receptors (nomenclature recommended by the NC‐IUPHAR subcommittee on pattern recognition receptors [18]) share a common domain organisation. This consists of an N‐terminal effector domain, a central nucleotide‐binding and oligomerization domain (NOD; also referred to as a NACHT domain), and C‐terminal leucine‐rich repeats (LRR) which have regulatory and ligand recognition functions. The type of effector domain has resulted in the division of NLR family members into two major sub‐families, NLRC and NLRP, along with three smaller sub‐families NLRA, NLRB and NLRX [178]. NLRC members express an N‐terminal caspase recruitment domain (CARD) and NLRP members an N‐terminal Pyrin domain (PYD).

Upon activation the NLRC family members NOD1 (NLRC1) and NOD2 (NLRC2) recruit a serine/threonine kinase *RIPK2* (receptor interacting serine/threonine kinase 2, O43353, also known as CARD3, CARDIAK, RICK, RIP2) leading to signalling through NF*κ*B and MAP kinase. Activation of NLRC4 (previously known as IPAF) and members of the NLRP3 family, including NLRP1 and NLRP3, leads to formation of a large multiprotein complex known as the inflammasome. In addition to NLR proteins other key members of the inflammasome include the adaptor protein ASC (apoptosis‐associated speck‐like protein containing a CARD, also known as *PYCARD*, CARD5, TMS1, Q9ULZ3) and inflammatory caspases. The inflammasome activates the pro‐inflammatory cytokines IL‐1*β* (*IL1B*, P01584) and IL‐18 (*IL18*, Q14116) [18, 37].

**Table:** 

Nomenclature	nucleotide‐binding oligomerizationdomain containing 1	nucleotide‐binding oligomerization domain containing 2	NLRC3	NLRC4	NLRC5
Common abreviation	NOD1	NOD2	–	–	–
HGNC, UniProt	*NOD1*, Q9Y239	*NOD2*, Q9HC29	*NLRC3*, Q7RTR2	*NLRC4*, Q9NPP4	*NLRC5*, Q86WI3
Agonists	meso‐DAP	muramyl dipeptide	–	–	–
Comments	–	NOD2 has also been reported to be activated by ssRNA [160] although this has not been widely reproduced.	–	NLRC4 forms an inflammasome in conjunction with the NAIP proteins and responds to bacterial flagellin and type III secretion system rod proteins.	–

**Table:** 

Nomenclature	NLRX1	CIITA	NLRP1	NLRP2
HGNC, UniProt	*NLRX1*, Q86UT6	*CIITA*, P33076	*NLRP1*, Q9C000	*NLRP2*, Q9NX02
Agonists	–	–	muramyl dipeptide	–
Comments	–	–	NLRP1 has 3 murine orthologues which lack the N‐terminal Pyrin domain. Murine NLRP1b (ENSMUSG00000070390) is the best characterised, responding to Anthrax Lethal Toxin.	Along with NLRP7, NLRP2 is the product of a primate‐specific gene duplication.

**Table:** 

Nomenclature	NLRP3	NLRP4	NLRP5	NLRP6
HGNC, UniProt	*NLRP3*, Q96P20	*NLRP4*, Q96MN2	*NLRP5*, P59047	*NLRP6*, P59044
Inhibitors	MCC950 (pIC_50_>8) [30]	–	–	–
Comments	Multiple virus particles have been shown to act as agonists, including Sendai and influenza. NLRP3 has been shown to be activated following disruption of cellular haemostasis by a wide‐variety of exogenous and endogenous molecules. The identity of the precise agonist that interacts with NLRP3 remains enigmatic.	Expanded in the mouse resulting in 7 orthologues.	–	–

**Table:** 

Nomenclature	NLRP7	NLRP8	NLRP9	NLRP10
HGNC, UniProt	*NLRP7*, Q8WX94	*NLRP8*, Q86W28	*NLRP9*, Q7RTR0	*NLRP10*, Q86W26
Comments	Absent in mouse. Along with NLRP2 the product of a primate‐specific gene duplication.	Absent in mouse	This receptor has three murine orthologues.	–

**Table:** 

Nomenclature	NLRP11	NLRP12	NLRP13	NLRP14
HGNC, UniProt	*NLRP11*, P59045	*NLRP12*, P59046	*NLRP13*, Q86W25	*NLRP14*, Q86W24
Comments	Absent in mouse	–	Absent in mouse	–

### Comments

NLRP3 has also been reported to respond to host‐derived products, known as danger‐associated molecular patterns, or DAMPs, including uric acid [122], ATP, L‐glucose, hyaluronan and amyloid *β* (*APP*, P05067) [163].

Loss‐of‐function mutations of NLRP3 are associated with cold autoinflammatory and Muckle‐Wells syndromes.

This family also includes NLR family, apoptosis inhibitory protein (*NAIP*, Q13075) which can be found in the 'Inhibitors of apoptosis (IAP) protein family' in the Other protein targets section of the Guide.

### Further Reading

Bryant CE *et al*. (2015) International Union of Basic and Clinical Pharmacology. XCVI. Pattern recognition receptors in health and disease. *Pharmacol. Rev.* **67**: 462‐504 [PMID:25829385]

## Receptor serine/threonine kinase (RSTK) family

### Overview

Receptor serine/threonine kinases (RTSK), EC 2.7.11.30, respond to particular cytokines, the transforming growth factor *β*(TGF*β*) and bone morphogenetic protein (BMP) families, and may be divided into two subfamilies on the basis of structural similarities. Agonist binding initiates formation of a cell‐surface complex of type I and type II RSTK, possibly heterotetrameric, where where both subunits express serine/threonine kinase activity. The type I receptor serine/threonine kinases are also known as activin receptors or activin receptor‐like kinases, ALKs, for which a systematic nomenclature has been proposed (ALK1‐7). The type II protein phosphorylates the kinase domain of the type I partner (sometimes referred to as the signal propagating subunit), causing displacement of the protein partners, such as the FKBP12 FK506‐binding protein *FKBP1A* (P62942) and allowing the binding and phosphorylation of particular members of the Smad family. These migrate to the nucleus and act as complexes to regulate gene transcription. Type III receptors, sometimes called co‐receptors or accessory proteins, regulate the signalling of the receptor complex, in either enhancing (for example, presenting the ligand to the receptor) or inhibitory manners. TGF*β* family ligand signalling may be inhibited by endogenous proteins, such as follistatin (*FST*, P19883), which binds and neutralizes activins to prevent activation of the target receptors.

Endogenous agonists, approximately 30 in man, are often described as paracrine messengers acting close to the source of production. They are characterized by six conserved cysteine residues and are divided into two subfamilies on the basis of sequence comparison and signalling pathways activated, the TGF*β*/activin/nodal subfamily and the BMP/GDF (growth/differentiation factor)/MIS (Müllerian inhibiting substance) subfamily. Ligands active at RSTKs appear to be generated as large precursors which undergo complex maturation processes [105]. Some are known to form disulphide‐linked homo‐ and/or heterodimeric complexes. Thus, inhibins are *α* subunits linked to a variety of *β* chains, while activins are combinations of *β* subunits.

### Comments

A number of endogenous inhibitory ligands have been identified for RSTKs, including BMP‐3 (*BMP3*, P12645), inhibin *α* (*INHA*, P05111), inhibin *β*C (*INHBC*, P55103) and inhibin *β*E (*INHBE*, P58166).

An appraisal of small molecule inhibitors of TGF*β* and BMP signalling concluded that TGF*β* pathway inhibitors were more selective than BMP signalling inhibitors [186]. The authors confirmed the selectivity of TGF‐beta RI inhibitor III to inhibit TGF*β* signalling through ALK4, ALK5, ALK7 [36]. Dorsomorphin inhibits BMP signalling through ALK2 and ALK3, it also inhibits AMP kinase [209].

**Smads** were identified as mammalian orthologues of Drosophila genes termed “mothers against decapentaplegic” and may be divided into Receptor‐regulated Smads (R‐Smads, including Smad1, Smad2, Smad3, Smad5 and Smad8), Co‐mediated Smad (Co‐Smad, Smad4) and Inhibitory Smads (I‐Smad, Smad6 and Smad7). R‐Smads form heteromeric complexes with Co‐Smad. I‐Smads compete for binding of R‐Smad with both receptors and Co‐Smad.

### Further Reading

Ehrlich M *et al*. (2011) Homomeric and heteromeric complexes among TGF‐*β* and BMP receptors and their roles in signaling. *Cell. Signal.* **23**: 1424‐32 [PMID:21515362]

Hinck AP. (2012) Structural studies of the TGF‐*β*s and their receptors ‐ insights into evolution of the TGF‐*β* superfamily. *FEBS Lett.* **586**: 1860‐70 [PMID:22651914]

Massagué J. (2012) TGF*β* signalling in context. *Nat. Rev. Mol. Cell Biol.* **13**: 616‐30 [PMID:22992590]

Moustakas A *et al*. (2009) The regulation of TGFbeta signal transduction. *Development* **136**: 3699‐714 [PMID:19855013]

Rider CC *et al*. (2010) Bone morphogenetic protein and growth differentiation factor cytokine families and their protein antagonists. *Biochem. J.* **429**: 1‐12 [PMID:20545624]

Santibañez JF *et al*. (2011) TGF‐*β*/TGF‐*β* receptor system and its role in physiological and pathological conditions. *Clin. Sci.* **121**: 233‐51 [PMID:21615335]

Xu P *et al*. (2012) Post‐translational regulation of TGF‐*β* receptor and Smad signaling. *FEBS Lett.* **586**: 1871‐84 [PMID:22617150]

## Type I receptor serine/threonine kinases

### Overview

The type I receptor serine/threonine kinases are also known as activin receptors or activin receptor‐like kinases, ALKs, for which a systematic nomenclature has been proposed (ALK1‐7).

**Table:** 

Nomenclature	activin A receptor type II‐like 1	activin A receptor, type I	bone morphogenetic protein receptor, type IA	activin A receptor, type IB
Common abreviation	ALK1	ALK2	BMPR1A	ALK4
HGNC, UniProt	*ACVRL1*, P37023	*ACVR1*, Q04771	*BMPR1A*, P36894	*ACVR1B*, P36896
EC number	2.7.11.30	2.7.11.30	2.7.11.30	2.7.11.30
Inhibitors	compound 13d [PMID: 23639540] (pIC_50_>8.3) [45], compound 13r [PMID: 23639540] (pIC_50_>8.3) [45]	compound 13d [PMID: 23639540] (pIC_50_>8.3) [45], ML347 (pIC_50_ 7.5) [45]	compound 13d [PMID: 23639540] (pIC_50_>8.3) [45]	–
Selective inhibitors	–	–	–	EW‐7197 (pIC_50_ 7.9) [82]

**Table:** 

Nomenclature	transforming growth factor, beta receptor 1	bone morphogenetic protein receptor, type IB	activin A receptor, type IC
Common abreviation	TGFBR1	BMPR1B	ALK7
HGNC, UniProt	*TGFBR1*, P36897	*BMPR1B*, O00238	*ACVR1C*, Q8NER5
EC number	2.7.11.30	2.7.11.30	2.7.11.30
Inhibitors	LY2109761 (p*K*_i_ 7.4) [125], compound 15b [PMID: 16539403] (pIC_50_ 7.1) [104]	compound 13d [PMID: 23639540] (pIC_50_>8.3) [45]	–
Selective inhibitors	EW‐7197 (pIC_50_ 8) [82]	–	–

## Type II receptor serine/threonine kinases

**Table:** 

Nomenclature	activin A receptor, type IIA	activin A receptor, type IIB	anti‐Mullerian hormone receptor, type II	bone morphogenetic protein receptor, type II (serine/threonine kinase)	transforming growth factor, beta receptor II (70/80kDa)
Common abreviation	ActR2	ActR2B	MISR2	BMPR2	TGFBR2
HGNC, UniProt	*ACVR2A*, P27037	*ACVR2B*, Q13705	*AMHR2*, Q16671	*BMPR2*, Q13873	*TGFBR2*, P37173
EC number	2.7.11.30	2.7.11.30	2.7.11.30	2.7.11.30	2.7.11.30
Inhibitors	–	–	–	–	compound 13d [PMID: 23639540] (pIC_50_ 7.6) [45]
Antibodies	–	bimagrumab (Binding) (p*K*_d_ 11.8) 12	–	–	–

## Type III receptor serine/threonine kinases

**Table:** 

Nomenclature	transforming growth factor, beta receptor III
Common abreviation	TGFBR3
HGNC, UniProt	*TGFBR3*, Q03167

## RSTK functional heteromers

### Overview

For the receptors listed on this page, the exact combination of subunits forming the functional heteromeric receptors is unknown.

**Table:** 

Nomenclature	Transforming growth factor *β* receptor	Bone morphogenetic protein receptors	Growth/differentiation factor receptors	Activin receptors	Anti‐Müllerian hormone receptors
Subunits	transforming growth factor, beta receptor 1 (Type I), transforming growth factor, beta receptor III (Type III), transforming growth factor, beta receptor II (70/80kDa) (Type II)	bone morphogenetic protein receptor, type IB (Type I), activin A receptor, type IIB (Type II), activin A receptor, type IIA (Type II), activin A receptor type II‐like 1 (Type I), activin A receptor, type I (Type I), bone morphogenetic protein receptor, type IA (Type I), bone morphogenetic protein receptor, type II (serine/threonine kinase) (Type II)	transforming growth factor, beta receptor 1 (Type I), bone morphogenetic protein receptor, type IB (Type I), activin A receptor, type IIB (Type II), activin A receptor, type IIA (Type II), activin A receptor, type IC (Type I), bone morphogenetic protein receptor, type IA (Type I), activin A receptor, type IB (Type I), bone morphogenetic protein receptor, type II (serine/threonine kinase) (Type II)	activin A receptor, type IIB (Type II), activin A receptor, type IIA (Type II), activin A receptor, type IC (Type I), activin A receptor, type IB (Type I)	anti‐Mullerian hormone receptor, type II (Type II), bone morphogenetic proteinreceptor, type IB (Type I), activin A receptor,type I (Type I), bone morphogenetic proteinreceptor, type IA (Type I)
Coupling	Smad2, Smad3 [134, 167]	Smad1, Smad5, Smad8 [134, 167]	Smad1, Smad5, Smad8 [134, 167]	Smad2, Smad3 [167]	Smad1, Smad5, Smad8 [134, 167]
Endogenous agonists	TGF*β*1 (*TGFB1*, P01137), TGF*β*2 (*TGFB2*, P61812), TGF*β*3 (*TGFB3*, P10600)	BMP‐10 (*BMP10*, O95393), BMP‐2 (*BMP2*, P12643), BMP‐4 (*BMP4*, P12644), BMP‐5 (*BMP5*, P22003), BMP‐6 (*BMP6*, P22004), BMP‐7 (*BMP7*, P18075), BMP‐8A (*BMP8A*, Q7Z5Y6), BMP‐8B (*BMP8B*, P34820), BMP‐9 (*GDF2*, Q9UK05)	growth/differentiation factor‐1 (*GDF1*, P27539), growth/differentiation factor‐10 (*GDF10*, P55107), growth/differentiation factor‐3 (*GDF3*, Q9NR23), growth/differentiation factor‐7 (*GDF7*, Q7Z4P5), growth/differentiation factor‐9 (*GDF9*, O60383)	activin A (*INHBA*, P08476), activin AB (*INHBA* *INHBB*, P08476 P09529), activin B (*INHBB*, P09529), inhibin A (*INHA* *INHBA*, P05111 P08476)	Müllerian inhibiting substance (*AMH*, P03971)
Comments	–	–	–	Activin receptors are heteromeric complexes comprising activin receptor type I and type II subunits.	–

## Receptor tyrosine kinases

### Overview

Receptor tyrosine kinases (RTKs), a family of cell‐surface receptors, which transduce signals to polypeptide and protein hormones, cytokines and growth factors are key regulators of critical cellular processes, such as proliferation and differentiation, cell survival and metabolism, cell migration and cell cycle control [14, 62, 184]. In the human genome, 58 RTKs have been identified, which fall into 20 families [102].

All RTKs display an extracellular ligand binding domain, a single transmembrane helix, a cytoplasmic region containing the protein tyrosine kinase activity (occasionally split into two domains by an insertion, termed the kinase insertion), with juxta‐membrane and C‐terminal regulatory regions. Agonist binding to the extracellular domain evokes dimerization, and sometimes oligomerization, of RTKs (a small subset of RTKs forms multimers even in the absence of activating ligand). This leads to autophosphorylation in the tyrosine kinase domain in a trans orientation, serving as a site of assembly of protein complexes and stimulation of multiple signal transduction pathways, including phospholipase C‐*γ*, mitogen‐activated protein kinases and phosphatidylinositol 3‐kinase [184].

RTKs are of widespread interest not only through physiological functions, but also as drug targets in many types of cancer and other disease states. Many diseases result from genetic changes or abnormalities that either alter the activity, abundance, cellular distribution and/or regulation of RTKs. Therefore, drugs that modify the dysregulated functions of these RTKs have been developed which fall into two categories. One group is often described as ‘biologicals’, which block the activation of RTKs directly or by chelating the cognate ligands, while the second are small molecules designed to inhibit the tyrosine kinase activity directly.

### Further Reading

Alsina FC *et al*. (2012) New insights into the control of neurotrophic growth factor receptor signaling: implications for nervous system development and repair. *J. Neurochem.* **123**: 652‐61 [PMID:22994539]

Arteaga CL *et al*. (2012) Treatment of HER2‐positive breast cancer: current status and future perspectives. *Nat Rev Clin Oncol* **9**: 16‐32 [PMID:22124364]

Camidge DR *et al*. (2012) Treating ALK‐positive lung cancer–early successes and future challenges. *Nat Rev Clin Oncol* **9**: 268‐77 [PMID:22473102]

Chen Y *et al*. (2012) Eph receptors at synapses: implications in neurodegenerative diseases. *Cell. Signal.* **24**: 606‐11 [PMID:22120527]

Fu HL *et al*. (2013) Discoidin domain receptors: unique receptor tyrosine kinases in collagen‐mediated signaling. *J. Biol. Chem.* **288**: 7430‐7 [PMID:23335507]

Gherardi E *et al*. (2012) Targeting MET in cancer: rationale and progress. *Nat. Rev. Cancer* **12**: 89‐103 [PMID:22270953]

Goetz R *et al*. (2013) Exploring mechanisms of FGF signalling through the lens of structural biology. *Nat. Rev. Mol. Cell Biol.* **14**: 166‐80 [PMID:23403721]

Guillemot F *et al*. (2011) From cradle to grave: the multiple roles of fibroblast growth factors in neural development. *Neuron* **71**: 574‐88 [PMID:21867876]

Higashiyama S *et al*. (2011) Ectodomain shedding and remnant peptide signalling of EGFRs and their ligands. *J. Biochem.* **150**: 15‐22 [PMID:21610047]

Ibáñez CF *et al*. (2012) p75 neurotrophin receptor signaling in nervous system injury and degeneration: paradox and opportunity. *Trends Neurosci.* **35**: 431‐40 [PMID:22503537]

Koh GY. (2013) Orchestral actions of angiopoietin‐1 in vascular regeneration. *Trends Mol Med* **19**: 31‐9 [PMID:23182855]

Larsen AK *et al*. (2011) Targeting EGFR and VEGF(R) pathway cross‐talk in tumor survival and angiogenesis. *Pharmacol. Ther.* **131**: 80‐90 [PMID:21439312]

Lefebvre J *et al*. (2012) Met degradation: more than one stone to shoot a receptor down. *FASEB J.* **26**: 1387‐99 [PMID:22223753]

Leitinger B. (2011) Transmembrane collagen receptors. *Annu. Rev. Cell Dev. Biol.* **27**: 265‐90 [PMID:21568710]

Lennartsson J *et al*. (2012) Stem cell factor receptor/c‐Kit: from basic science to clinical implications. *Physiol. Rev.* **92**: 1619‐49 [PMID:23073628]

Liang G *et al*. (2012) Anticancer molecules targeting fibroblast growth factor receptors. *Trends Pharmacol. Sci.* **33**: 531‐41 [PMID:22884522]

Lisle JE *et al*. (2013) Eph receptors and their ligands: promising molecular biomarkers and therapeutic targets in prostate cancer. *Biochim. Biophys. Acta* **1835**: 243‐57 [PMID:23396052]

Lu B *et al*. (2013) BDNF‐based synaptic repair as a disease‐modifying strategy for neurodegenerative diseases. *Nat. Rev. Neurosci.* **14**: 401‐16 [PMID:23674053]

Morandi A *et al*. (2011) RET in breast cancer: functional and therapeutic implications. *Trends Mol Med* **17**: 149‐57 [PMID:21251878]

Peters S *et al*. (2012) MET: a promising anticancer therapeutic target. *Nat Rev Clin Oncol* **9**: 314‐26 [PMID:22566105]

Roskoski Jr R. (2013) Anaplastic lymphoma kinase (ALK): structure, oncogenic activation, and pharmacological inhibition. *Pharmacol. Res.* **68**: 68‐94 [PMID:23201355]

Sheffler‐Collins SI *et al*. (2012) EphBs: an integral link between synaptic function and synaptopathies. *Trends Neurosci.* **35**: 293‐304 [PMID:22516618]

Shibuya M. (2013) Vascular endothelial growth factor and its receptor system: physiological functions in angiogenesis and pathological roles in various diseases. *J. Biochem.* **153**: 13‐9 [PMID:23172303]

Turner CA *et al*. (2012) The fibroblast growth factor family: neuromodulation of affective behavior. *Neuron* **76**: 160‐74 [PMID:23040813]

Woo KV *et al*. (2011) Role of Tie1 in shear stress and atherosclerosis. *Trends Cardiovasc. Med.* **21**: 118‐23 [PMID:22681967]

Yamanashi Y *et al*. (2012) Activation of receptor protein‐tyrosine kinases from the cytoplasmic compartment. *J. Biochem.* **151**: 353‐9 [PMID:22343747]

## Type I RTKs: ErbB (epidermal growth factor) receptor family

### Overview

ErbB family receptors are Class I receptor tyrosine kinases [62]. ERBB2 (also known as HER‐2 or NEU) appears to act as an essential partner for the other members of the family without itself being activated by a cognate ligand [63]. Ligands of the ErbB family of receptors are peptides, many of which are generated by proteolytic cleavage of cell‐surface proteins. HER/ErbB is the viral counterpart to the receptor tyrosine kinase EGFR. All family members heterodimerize with each other to activate downstream signalling pathways and are aberrantly expressed in many cancers, particularly forms of breast cancer.

**Table:** 

Nomenclature	epidermal growth factor receptor	erb‐b2 receptor tyrosine kinase 2	erb‐b2 receptor tyrosine kinase 3	erb‐b2 receptor tyrosine kinase 4
Common abreviation	EGFR	HER2	HER3	HER4
HGNC, UniProt	*EGFR*, P00533	*ERBB2*, P04626	*ERBB3*, P21860	*ERBB4*, Q15303
EC number	2.7.10.1	2.7.10.1	2.7.10.1	2.7.10.1
Endogenous ligands	EGF (*EGF*, P01133) (Binding), HB‐EGF (*HBEGF*, Q99075) (Binding), TGF*α* (*TGFA*, P01135) (Binding), amphiregulin (*AREG*, P15514) (Binding), betacellulin (*BTC*, P35070) (Binding), epigen (*EPGN*, Q6UW88) (Binding), epiregulin (*EREG*, O14944) (Binding)	–	neuregulin‐1 (*NRG1*, Q02297), neuregulin‐2 (*NRG2*, O14511)	HB‐EGF (*HBEGF*, Q99075), betacellulin (*BTC*, P35070), epiregulin (*EREG*, O14944), neuregulin‐1 (*NRG1*, Q02297), neuregulin‐2 (*NRG2*, O14511), neuregulin‐3 (*NRG3*, P56975), neuregulin‐4 (*NRG4*, Q8WWG1)
Inhibitors	canertinib (p*K*_d_ 9.7) [38], afatinib (p*K*_d_ 9.6) [38], XL‐647 (pIC_50_ 9.5) [57], afatinib (pIC_50_ 8–9.3) [33, 103], erlotinib (p*K*_d_ 9.2) [38], erlotinib (pIC_50_ 9) [207], gefitinib (p*K*_d_ 9) [38], canertinib (pIC_50_ 8.8) [170], BMS‐690514 (pIC_50_ 8.3) [117], gefitinib (p*K*_i_ 8.3) [197], AG1478 (pIC_50_ 8.2) [181], poziotinib (pIC_50_ 8.1) [140], lapatinib (pIC_50_ 8) [159], EGFR/ErbB‐2 inhibitor (pIC_50_ 7.7) [28], AG 112 (pIC_50_ 6.9) [56], rociletinib (p*K*_i_ 6.5) [187], AG 490 (pIC_50_ 6.4) [55]	poziotinib (pIC_50_ 8.3) [140], neratinib (p*K*_d_ 8.2) [38], lapatinib (p*K*_d_ 8.1) [38], lapatinib (pIC_50_ 8) [159], CP‐724714 (pIC_50_ 7.9) [65], XL‐647 (pIC_50_ 7.8) [57], BMS‐690514 (pIC_50_ 7.7) [117], neratinib (pIC_50_ 7.2) [155], EGFR/ErbB‐2 inhibitor (pIC_50_ 7.1) [28]	–	poziotinib (pIC_50_ 7.6) [140]
Antibodies	necitumumab (Binding) (p*K*_d_ 9.5) 111, cetuximab (Binding) (p*K*_d_ 9.4) 60, panitumumab (Inhibition)	pertuzumab (Inhibition) (pIC_50_>8) 84, trastuzumab (Inhibition)	–	–

### Comments

[^125^I]EGF (human) has been used to label the ErbB1 EGF receptor. The extracellular domain of ErbB2 can be targetted by the antibodies trastuzumab and pertuzumab to inhibit ErbB family action. The intracellular ATP‐binding site of the tyrosine kinase domain can be inhibited by GW583340 (7.9‐8.0, [54]), gefitinib, erlotinib and tyrphostins AG879 and AG1478.

## Type II RTKs: Insulin receptor family

### Overview

The circulating peptide hormones insulin (*INS*, P01308) and the related insulin‐like growth factors (IGF) activate Class II receptor tyrosine kinases [62], to evoke cellular responses, mediated through multiple intracellular adaptor proteins. Exceptionally amongst the catalytic receptors, the functional receptor in the insulin receptor family is derived from a single gene product, cleaved post‐translationally into two peptides, which then cross‐link via disulphide bridges to form a heterotetramer. Intriguingly, the endogenous peptide ligands are formed in a parallel fashion with post‐translational processing producing a heterodimer linked by disulphide bridges. Signalling through the receptors is mediated through a rapid autophosphorylation event at intracellular tyrosine residues, followed by recruitment of multiple adaptor proteins, notably *IRS1* (P35568), *IRS2* (Q9Y4H2), *SHC1* (P29353), *GRB2* (P62993) and *SOS1* (Q07889).

Serum levels of free IGFs are kept low by the action of IGF binding proteins (IGFBP1‐5, P08833, P18065, P17936, P22692, P24593), which sequester the IGFs; overexpression of IGFBPs may induce apoptosis, while IGFBP levels are also altered in some cancers.

**Table:** 

Nomenclature	Insulin receptor	Insulin‐like growth factor I receptor	Insulin receptor‐related receptor
Common abreviation	InsR	IGF1R	IRR
HGNC, UniProt	*INSR*, P06213	*IGF1R*, P08069	*INSRR*, P14616
EC number	2.7.10.1	2.7.10.1	2.7.10.1
Inhibitors	–	BMS‐754807 (pIC_50_ 8.7) [199], GSK‐1838705A (pIC_50_ 8.7) [161], GSK‐1838705A (p*K*_d_ 8.1) [38], PQ401 (pIC_50_>6) [50], AG 1024 (pIC_50_ 4.7) [153]	–
Selective inhibitors	–	NVP‐AEW541 (pIC_50_ 9.4) [53]	–
Endogenous agonists	insulin (*INS*, P01308)	insulin‐like growth factor 1 (*IGF1*, P05019), insulin‐like growth factor 2 (*IGF2*, P01344)	–

### Comments

There is evidence for low potency binding and activation of insulin receptors by IGF1. IGF2 also binds and activates the cation‐independent mannose 6‐phosphate receptor (also known as the insulin‐like growth factor II receptor), which lacks classical signalling capacity and appears to subserve a trafficking role [115]. INSRR, which has a much more discrete localization, being predominant in the kidney [95], currently lacks a cognate ligand or evidence for functional impact.

Antibodies targetting IGF1, IGF2 and the extracellular portion of the IGF1 receptor are in clinical trials.

PQ401 inhibits the insulin‐like growth factor receptor [5], while BMS‐536924 inhibits both the insulin receptor and the insulin‐like growth factor receptor [198].

## Type III RTKs: PDGFR, CSFR, Kit, FLT3 receptor family

### Overview

Type III RTKs include PDGFR, CSF‐1R (Ems), Kit and FLT3, which function as homo‐ or heterodimers. Endogenous ligands of PDGF receptors are homo‐ or heterodimeric: PDGFA, PDGFB, VEGFE and PDGFD (*PDGFD*, Q9GZP0) combine as homo‐ or heterodimers to activate homo‐ or heterodimeric PDGF receptors. SCF is a dimeric ligand for KIT. Ligands for CSF1R are either monomeric or dimeric glycoproteins, while the endogenous agonist for FLT3 is a homodimer.

**Table:** 

Nomenclature	platelet‐derived growth factor receptor, alpha polypeptide	platelet‐derived growth factor receptor, beta polypeptide	v‐kit Hardy‐Zuckerman 4 feline sarcoma viral oncogene homolog	colony stimulating factor 1 receptor	fms‐related tyrosine kinase 3
Common abreviation	PDGFR*α*	PDGFR*β*	Kit	CSFR	FLT3
HGNC, UniProt	*PDGFRA*, P16234	*PDGFRB*, P09619	*KIT*, P10721	*CSF1R*, P07333	*FLT3*, P36888
EC number	2.7.10.1	2.7.10.1	2.7.10.1	2.7.10.1	2.7.10.1
Endogenous ligands	PDGF	PDGF	–	–	–
Endogenous ligands	–	–	stem cell factor (*KITLG*, P21583)	G‐CSF (*CSF3*, P09919), GM‐CSF (*CSF2*, P04141), M‐CSF (*CSF1*, P09603)	Fms‐related tyrosine kinase 3 ligand (*FLT3LG*, P49771)
Inhibitors	PP121 (pIC_50_ 8.7) [7], crenolanib (p*K*_d_ 8.7) [71], ENMD‐2076 (pIC_50_ 7.2) [149]	crenolanib (p*K*_d_ 8.5) [71], SU‐14813 (pIC_50_ 8.4) [147], famitinib (pIC_50_ 8.4) [24], sunitinib (pIC_50_ 8.2) [91], sunitinib (p*K*_i_ 8.1) [126]	sunitinib (p*K*_d_ 9.4) [38], famitinib (pIC_50_ 8.7) [24], masitinib (p*K*_d_ 8.1) [38], SU‐14813 (pIC_50_ 7.8) [147], AKN‐028 (pIC_50_ 7.5) [46], sorafenib (pIC_50_ 7.2) [196]	JNJ‐28312141 (pIC_50_ 9.2) [116], Ki‐20227 (p*K*_d_ 9.1) [38], Ki‐20227 (pIC_50_ 8.7) [143], GW‐2580 (p*K*_d_ 8.7) [38], JNJ‐28312141 (p*K*_d_ 8.5) [38]	AC710 (p*K*_d_ 9.3) [109], linifanib (p*K*_d_ 9.2) [38], dovitinib (p*K*_d_ 9.2) [38], crenolanib (p*K*_d_ 9.1) [71], AST‐487 (p*K*_d_ 9.1) [38], compound 8h [PMID: 22765894] (pIC_50_ 9.1) [88], dovitinib (pIC_50_ 8.5–9) [157, 183], ENMD‐2076 (pIC_50_ 8.5) [149], tandutinib (p*K*_d_ 8.5) [38], quizartinib (pIC_50_ 8.4) [206], AKN‐028 (pIC_50_ 8.2) [46], KW‐2449 (pIC_50_ 8.2) [168], lestaurtinib (p*K*_d_ 8.1) [38], midostaurin (p*K*_d_ 8) [38], KW‐2449 (p*K*_d_ 7.8) [38], sorafenib (pIC_50_ 7.2) [196], AST‐487 (p*K*_i_ 6.9) [193], tandutinib (pIC_50_ 6.7) [86], AST‐487 (pIC_50_ 6.3) [2], midostaurin (pIC_50_ 6.3) [192]
Selective inhibitors	CP‐673451 (pIC_50_ 8) [158]	CP‐673451 (pIC_50_ 9) [158]	–	GW‐2580 (pIC_50_ 7.2) [32]	G749 (pIC_50_ 9.4) [99]
Comments	–	–	–	–	5'‐fluoroindirubinoxime has been described as a selective FLT3 inhibitor [25].

### Comments

Various small molecular inhibitors of type III RTKs have been described, including imatinib and nilotinib (targetting PDGFR, KIT and CSF1R); midostaurin and AC220 (quizartinib; FLT3), as well as pan‐type III RTK inhibitors such as sunitinib and sorafenib [148]; 5'‐fluoroindirubinoxime has been described as a selective FLT3 inhibitor [2].

## Type IV RTKs: VEGF (vascular endothelial growth factor) receptor family

### Overview

VEGF receptors are homo‐ and heterodimeric proteins, which are characterized by seven Ig‐like loops in their extracellular domains and a split kinase domain in the cytoplasmic region. They are key regulators of angiogenesis and lymphangiogenesis; as such, they have been the focus of drug discovery for conditions such as metastatic cancer. Splice variants of VEGFR1 and VEGFR2 generate truncated proteins limited to the extracellular domains, capable of homodimerisation and binding VEGF ligands as a soluble, non‐signalling entity. Ligands at VEGF receptors are typically homodimeric. VEGFA (*VEGFA*, P15692) is able to activate VEGFR1 homodimers, VEGFR1/2 heterodimers and VEGFR2/3 heterodimers. VEGFB (*VEGFB*, P49765) and placental growth factor (*PGF*, P49763) activate VEGFR1 homodimers, while VEGFC (*VEGFC*, P49767) and VEGFD (*FIGF*, O43915) activate VEGFR2/3 heterodimers and VEGFR3 homodimers, and, following proteolysis, VEGFR2 homodimers.

**Table:** 

Nomenclature	fms‐related tyrosine kinase 1	kinase insert domain receptor	fms‐related tyrosine kinase 4
Common abreviation	VEGFR‐1	VEGFR‐2	VEGFR‐3
HGNC, UniProt	*FLT1*, P17948	*KDR*, P35968	*FLT4*, P35916
EC number	2.7.10.1	2.7.10.1	2.7.10.1
Endogenous ligands	VEGFA (*VEGFA*, P15692), VEGFB (*VEGFB*, P49765)	VEGFA (*VEGFA*, P15692), VEGFC (*VEGFC*, P49767), VEGFE (*PDGFC*, Q9NRA1)	VEGFC (*VEGFC*, P49767), VEGFD (*FIGF*, O43915), VEGFE (*PDGFC*, Q9NRA1)
Inhibitors	SU‐14813 (pIC_50_ 8.7) [147], CEP‐11981 (pIC_50_ 8.5) [77], semaxanib (pIC_50_ 8.1) [15]	axitinib (pIC_50_ 9.6) [100], cabozantinib (pIC_50_ 9.5) [203], foretinib (pIC_50_ 8.2–9.1) [137], cediranib (p*K*_d_ 9) [38], XL‐647 (pIC_50_ 8.8) [57], compound 13a [PMID: 23639540] (pIC_50_ 8.8) [45], SU‐14813 (p*K*_d_ 8.6) [38], motesanib (p*K*_d_ 8.6) [38], famitinib (pIC_50_ 8.3) [24], axitinib (p*K*_d_ 8.2) [38], PLX‐4720 (p*K*_i_ 8.1) [126], CP‐547632 (pIC_50_ 8) [11], PP121 (pIC_50_ 7.9) [7], golvatinib (pIC_50_ 7.8) [139], brivanib (pIC_50_ 7.6) [13], ENMD‐2076 (pIC_50_ 7.4) [149], BMS‐690514 (pIC_50_ 7.3) [117], SU‐14813 (pIC_50_ 7.3) [147], sorafenib (p*K*_d_ 7.2) [38], vatalanib (p*K*_d_ 7.2) [38], sorafenib (pIC_50_ 7.1) [196]	XL‐647 (pIC_50_ 8.1) [57], sunitinib (pIC_50_ 8.1) [87], nintedanib (pIC_50_ 7.9) [74]
(Sub)family‐selective inhibitors	pazopanib (pIC_50_ 8) [68]	pazopanib (p*K*_d_ 7.8) [38], pazopanib (pIC_50_ 7.5) [68]	pazopanib (pIC_50_ 7.3) [68]
Antibodies	–	ramucirumab (Antagonist) (pIC_50_ 9) [113]	–

### Comments

The VEGFR, as well as VEGF ligands, have been targeted by antibodies and tyrosine kinase inhibitors. DMH4 [49], Ki8751 [94] and ZM323881, a novel inhibitor of vascular endothelial growth factor‐receptor‐2 tyrosine kinase activity [195] are described as VEGFR2‐selective tyrosine kinase inhibitors. Bevacizumab is a monoclonal antibody directed against VEGF‐A, used clinically for the treatment of certain metastatic cancers; an antibody fragment has been used for wet age‐related macular degeneration.

## Type V RTKs: FGF (fibroblast growth factor) receptor family

### Overview

Fibroblast growth factor (FGF) family receptors act as homo‐ and heterodimers, and are characterized by Ig‐like loops in the extracellular domain, in which disulphide bridges may form across protein partners to allow the formation of covalent dimers which may be constitutively active. FGF receptors have been implicated in achondroplasia, angiogenesis and numerous congenital disorders. At least 22 members of the FGF gene family have been identified in the human genome [11]. Within this group, subfamilies of FGF may be divided into canonical, intracellular and hormone‐like FGFs. FGF1‐FGF10 have been identified to act through FGF receptors, while FGF11‐14 appear to signal through intracellular targets. Other family members are less well characterized [194].

**Table:** 

Nomenclature	fibroblast growth factor receptor 1	fibroblast growth factor receptor 2	fibroblast growth factor receptor 3	fibroblast growth factor receptor 4
Common abreviation	FGFR1	FGFR2	FGFR3	FGFR4
HGNC, UniProt	*FGFR1*, P11362	*FGFR2*, P21802	*FGFR3*, P22607	*FGFR4*, P22455
EC number	2.7.10.1	2.7.10.1	2.7.10.1	2.7.10.1
Endogenous ligands	FGF‐1 (*FGF1*, P05230), FGF‐2 (*FGF2*, P09038), FGF‐4 (*FGF4*, P08620) >FGF‐5 (*FGF5*, P12034), FGF‐6 (*FGF6*, P10767) [145]	FGF‐1 (*FGF1*, P05230) >FGF‐4 (*FGF4*, P08620), FGF‐7 (*FGF7*, P21781), FGF‐9 (*FGF9*, P31371) >FGF‐2 (*FGF2*, P09038), FGF‐6 (*FGF6*, P10767) [145]	FGF‐1 (*FGF1*, P05230), FGF‐2 (*FGF2*, P09038), FGF‐9 (*FGF9*, P31371) >FGF‐4 (*FGF4*, P08620), FGF‐8 (*FGF8*, P55075) [145]	FGF‐1 (*FGF1*, P05230), FGF‐2 (*FGF2*, P09038), FGF‐4 (*FGF4*, P08620), FGF‐9 (*FGF9*, P31371) >FGF‐6 (*FGF6*, P10767), FGF‐8 (*FGF8*, P55075) [145]
(Sub)family‐selective inhibitors	LY2874455 (pIC_50_ 8.6) [208]	LY2874455 (pIC_50_ 8.6) [208]	LY2874455 (pIC_50_ 8.2) [208]	LY2874455 (pIC_50_ 8.2) [208]
Agonists	–	palifermin	–	–

### Comments

Splice variation of the receptors can influence agonist responses. *FGFRL1* (Q8N441) is a truncated kinase‐null analogue.

Various antibodies and tyrosine kinase inhibitors have been developed against FGF receptors [107, 210]. PD161570 is an FGFR tyrosine kinase inhibitor [10], while PD173074 has been described to inhibit FGFR1 and FGFR3 [169].

## Type VI RTKs: PTK7/CCK4

### Overview

The PTK7 receptor is associated with polarization of epithelial cells and the development of neural structures. Sequence analysis suggests that the gene product is catalytically inactive as a protein kinase, although there is evidence for a role in Wnt signalling [152].

**Table:** 

Nomenclature	protein tyrosine kinase 7 (inactive)
Common abreviation	CCK4
HGNC, UniProt	*PTK7*, Q13308
EC number	2.7.10.1

### Comments

Thus far, no selective PTK7 inhibitors have been described.

## Type VII RTKs: Neurotrophin receptor/Trk family

### Overview

The neurotrophin receptor family of RTKs include trkA, trkB and trkC (tropomyosin‐related kinase) receptors, which respond to NGF, BDNF and neurotrophin‐3, respectively. They are associated primarily with proliferative and migration effects in neural systems. Various isoforms of neurotrophin receptors exist, including truncated forms of trkB and trkC, which lack catalytic domains. p75(TNFRSF16, also known as nerve growth factor receptor), which has homologies with tumour necrosis factor receptors, lacks a tyrosine kinase domain, but can signal via ceramide release and nuclear factor *κ*B (NF‐*κ*B) activation. Both trkA and trkB contain two leucine‐rich regions and can exist in monomeric or dimeric forms.

**Table:** 

Nomenclature	neurotrophic tyrosine kinase, receptor, type 1	neurotrophic tyrosine kinase, receptor, type 2	neurotrophic tyrosine kinase, receptor, type 3
Common abreviation	trkA	trkB	trkC
HGNC, UniProt	*NTRK1*, P04629	*NTRK2*, Q16620	*NTRK3*, Q16288
EC number	2.7.10.1	2.7.10.1	2.7.10.1
Endogenous ligands	NGF (*NGF*, P01138) >neurotrophin‐3 (*NTF3*, P20783)	BDNF (*BDNF*, P23560), neurotrophin‐4 (*NTF4*, P34130) >neurotrophin‐3 (*NTF3*, P20783)	–
Endogenous ligands	–	–	neurotrophin‐3 (*NTF3*, P20783)
Inhibitors	compound 2c [PMID: 24900538] (pIC_50_ 8.9) [189], milciclib (pIC_50_ 7.3) [17]	–	–
(Sub)family‐selective inhibitors	AZD1332 (pIC_50_>8.3) 9, GNF‐5837 (pIC_50_ 8) [5]	AZD1332 (pIC_50_>8.3) 9, GNF‐5837 (pIC_50_ 8.1) [5]	AZD1332 (pIC_50_>8.3) 9, GNF‐5837 (pIC_50_ 8.1) [5]

### Comments

[^125^I]NGF (human) and [^125^I]BDNF (human) have been used to label the trkA and trkB receptor, respectively. p75 influences the binding of NGF (*NGF*, P01138) and neurotrophin‐3 (*NTF3*, P20783) to trkA. The ligand selectivity of p75 appears to be dependent on the cell type; for example, in sympathetic neurones, it binds neurotrophin‐3 (*NTF3*, P20783) with comparable affinity to trkC [40].

Small molecule agonists of trkB have been described, including LM22A4 [123], while ANA12 has been described as a non‐competitive antagonist of BDNF binding to trkB [23]. GNF5837 is a family‐selective tyrosine kinase inhibitor [4], while the tyrosine kinase activity of the trkA receptor can be inhibited by GW441756 (p_IC_50= 8.7, [200]) and tyrphostin AG879 [142].

## Type VIII RTKs: ROR family

### Overview

Members of the ROR family appear to be activated by ligands complexing with other cell‐surface proteins. Thus, ROR1 and ROR2 appear to be activated by Wnt‐5a (*WNT5A*, P41221) binding to a Frizzled receptor thereby forming a cell‐surface multiprotein complex [64].

**Table:** 

Nomenclature	receptor tyrosine kinase‐like orphan receptor 1	receptor tyrosine kinase‐like orphan receptor 2
Common abreviation	ROR1	ROR2
HGNC, UniProt	*ROR1*, Q01973	*ROR2*, Q01974
EC number	2.7.10.1	2.7.10.1

## Type IX RTKs: MuSK

### Overview

The muscle‐specific kinase MuSK is associated with the formation and organisation of the neuromuscular junction from the skeletal muscle side. Agrin (*AGRN*, O00468) forms a complex with low‐density lipoprotein receptor‐related protein 4 (*LRP4*, O75096) to activate MuSK [89].

**Table:** 

Nomenclature	muscle, skeletal, receptor tyrosine kinase
Common abreviation	MuSK
HGNC, UniProt	*MUSK*, O15146
EC number	2.7.10.1

### Comments

Thus far, no selective MuSK inhibitors have been described.

## Type X RTKs: HGF (hepatocyte growth factor) receptor family

### Overview

HGF receptors regulate maturation of the liver in the embryo, as well as having roles in the adult, for example, in the innate immune system. HGF is synthesized as a single gene product, which is post‐translationally processed to yield a heterodimer linked by a disulphide bridge. The maturation of HGF is enhanced by a serine protease, HGF activating complex, and inhibited by HGF‐inhibitor 1 (*SPINT1*, O43278), a serine protease inhibitor. MST1, the ligand of RON, is two disulphide‐linked peptide chains generated by proteolysis of a single gene product.

**Table:** 

Nomenclature	MET proto‐oncogene, receptor tyrosine kinase	macrophage stimulating 1 receptor
Common abreviation	MET	Ron
HGNC, UniProt	*MET*, P08581	*MST1R*, Q04912
EC number	2.7.10.1	2.7.10.1
Endogenous ligands	hepatocyte growth factor (*HGF*, P14210)	macrophage stimulating protein 1 (*MST1*, P09603)
Inhibitors	capmatinib (pIC_50_ 9.9) [112], SGX‐523 (p*K*_d_ 9.7) [38], PHA‐665752 (p*K*_d_ 9.6) [38], foretinib (pIC_50_ 9.3–9.4) [106, 137], cabozantinib (pIC_50_ 8.9) [203], foretinib (p*K*_d_ 8.9) [38], MK‐2461 (pIC_50_ 8.6) [146], BMS‐777607 (pIC_50_ 8.4) [164], PHA‐665752 (p*K*_i_ 8.4) [26], SU11274 (pIC_50_ 8) [190], golvatinib (pIC_50_ 7.8) [139], tivantinib (p*K*_i_ 6.4) [135]	BMS‐777607 (pIC_50_ 8.7) [164]
Selective inhibitors	SGX‐523 (pIC_50_ 8.4) [19]	–

### Comments

PF04217903 is a selective Met tyrosine kinase inhibitor [34].SU11274 is an inhibitor of the HGF receptor [162], with the possibility of further targets [8].

## Type XI RTKs: TAM (TYRO3‐, AXL‐ and MER‐TK) receptor family

### Overview

Members of this RTK family represented a novel structural motif, when sequenced. The ligands for this family, growth arrest specific protein 6 (*GAS6*, Q14393) and protein S (*PROS1*, P07225), are secreted plasma proteins which undergo vitamin K‐dependent post‐translational modifications generating carboxyglutamate‐rich domains which are able to bind to negatively‐charged surfaces of apoptotic cells.

**Table:** 

Nomenclature	AXL receptor tyrosine kinase	TYRO3 protein tyrosine kinase	MER proto‐oncogene, tyrosine kinase
Common abreviation	Axl	Tyro3	Mer
HGNC, UniProt	*AXL*, P30530	*TYRO3*, Q06418	*MERTK*, Q12866
EC number	2.7.10.1	2.7.10.1	2.7.10.1
Endogenous ligands	growth arrest specific protein 6 (*GAS6*, Q14393) [138], protein S (*PROS1*, P07225) [172]	growth arrest specific protein 6 (*GAS6*, Q14393) [138], protein S (*PROS1*, P07225) [172]	growth arrest specific protein 6 (*GAS6*, Q14393) [138]

### Comments

AXL tyrosine kinase inhibitors have been described [130].

## Type XII RTKs: TIE family of angiopoietin receptors

### Overview

The TIE family were initially associated with formation of blood vessels. Endogenous ligands are angiopoietin‐1 (*ANGPT1*, Q15389), angiopoietin‐2 (*ANGPT2*, O15123), and angiopoietin‐4 (*ANGPT4*, Q9Y264). Angiopoietin‐2 (*ANGPT2*, O15123) appears to act as an endogenous antagonist of angiopoietin‐1 function.

**Table:** 

Nomenclature	tyrosine kinase with immunoglobulin‐like and EGF‐like domains 1	TEK tyrosine kinase, endothelial
Common abreviation	TIE1	TIE2
HGNC, UniProt	*TIE1*, P35590	*TEK*, Q02763
EC number	2.7.10.1	2.7.10.1
Endogenous ligands	–	angiopoietin‐1 (*ANGPT1*, Q15389), angiopoietin‐4 (*ANGPT4*, Q9Y264)

## Type XIII RTKs: Ephrin receptor family

### Overview

Ephrin receptors are a family of 15 RTKs (the largest family of RTKs) with two identified subfamilies (EphA and EphB), which have a role in the regulation of neuronal development, cell migration, patterning and angiogenesis. Their ligands are membrane‐associated proteins, thought to be glycosylphosphatidylinositol‐linked for EphA (ephrin‐A1 (*EFNA1*, P20827) , ephrin‐A2 (*EFNA2*, O43921), ephrin‐A3 (*EFNA3*, P52797), ephrin‐A4 (*EFNA4*, P52798) and ephrin‐A5 (*EFNA5*, P52803)) and 1TM proteins for Ephrin B (ENSFM00250000002014: ephrin‐B1 (*EFNB1*, P98172), ephrin‐B2 (*EFNB2*, P52799) and ephrin‐B3 (*EFNB3*, Q15768)), although the relationship between ligands and receptors has been incompletely defined.

**Table:** 

Nomenclature	EPH receptor A1	EPH receptor A2	EPH receptor A3	EPH receptor A4	EPH receptor A5	EPH receptor A6	EPH receptor A7
Common abreviation	EphA1	EphA2	EphA3	EphA4	EphA5	EphA6	EphA7
HGNC, UniProt	*EPHA1*, P21709	*EPHA2*, P29317	*EPHA3*, P29320	*EPHA4*, P54764	*EPHA5*, P54756	*EPHA6*, Q9UF33	*EPHA7*, Q15375
EC number	2.7.10.1	2.7.10.1	2.7.10.1	2.7.10.1	2.7.10.1	2.7.10.1	2.7.10.1
Inhibitors	compound 20[PMID: 23489211] (pIC_50_ 5.6) [79]	–	–	–	–	–	–

**Table:** 

Nomenclature	EPH receptor A8	EPH receptor A10	EPH receptor B1	EPH receptor B2	EPH receptor B3	EPH receptor B4	EPH receptor B6
Common abreviation	EphA8	EphA10	EphB1	EphB2	EphB3	EphB4	EphB6
HGNC, UniProt	*EPHA8*, P29322	*EPHA10*, Q5JZY3	*EPHB1*, P54762	*EPHB2*, P29323	*EPHB3*, P54753	*EPHB4*, P54760	*EPHB6*, O15197
EC number	2.7.10.1	2.7.10.1	2.7.10.1	2.7.10.1	2.7.10.1	2.7.10.1	2.7.10.1
Inhibitors	–	–	compound 66[PMID: 19788238] (pIC_50_ 9) [97]	–	–	XL‐647 (pIC_50_ 8.9) [57]	–

## Type XIV RTKs: RET

### Overview

Ret proto‐oncogene (Rearranged during transfection) is a transmembrane tyrosine kinase enzyme which is employed as a signalling partner for members of the GDNF family receptors. Ligand‐activated GFR appears to recruit Ret as a dimer, leading to activation of further intracellular signalling pathways. Ret appears to be involved in neural crest development, while mutations may be involved in multiple endocrine neoplasia, Hirschsprung's disease, and medullary thyroid carcinoma.

**Table:** 

Nomenclature	ret proto‐oncogene
Common abreviation	Ret
HGNC, UniProt	*RET*, P07949
EC number	2.7.10.1
Inhibitors	tamatinib (pIC_50_ 8.3) [27], vandetanib (p*K*_d_ 7.5) [38], vandetanib (pIC_50_ 7) [22]

### Comments

A number of tyrosine kinase inhibitors targeting RET have been described [47].

## Type XV RTKs: RYK

### Overview

The ‘related to tyrosine kinase receptor’ (Ryk) is structurally atypical of the family of RTKs, particularly in the activation and ATP‐binding domains. RYK has been suggested to lack kinase activity and appears to be involved, with FZD8, in the Wnt signalling system [152].

**Table:** 

Nomenclature	receptor‐like tyrosine kinase
Common abreviation	RYK
HGNC, UniProt	*RYK*, P34925
EC number	2.7.10.1

### Comments

Thus far, no selective RYK inhibitors have been described.

## Type XVI RTKs: DDR (collagen receptor) family

### Overview

Discoidin domain receptors 1 and 2 (DDR1 and DDR2) are structurally‐related membrane protein tyrosine kinases activated by collagen. Collagen is probably the most abundant protein in man, with at least 29 families of genes encoding proteins, which undergo splice variation and post‐translational processing, and may exist in monomeric or polymeric forms, producing a triple‐stranded, twine‐like structure. In man, principal family members include COL1A1 (*COL1A1*, P02452), COL2A1 (*COL2A1*, P02458), COL3A1 (*COL3A1*, P02461) and COL4A1 (*COL4A1*, P02462).

**Table:** 

Nomenclature	discoidin domain receptor tyrosine kinase 1	discoidin domain receptor tyrosine kinase 2
Common abreviation	DDR1	DDR2
HGNC, UniProt	*DDR1*, Q08345	*DDR2*, Q16832
EC number	2.7.10.1	2.7.10.1
Inhibitors	compound 7k [PMID: 23521020] (pIC_50_ 8.6) [51]	–

### Comments

The tyrosine kinase inhibitors of DDR, imatinib and nilotinib, were identified from proteomic analysis [39]. Other collagen receptors include glycoprotein VI (Q9HCN6), leukocyte‐associated immunoglobulin‐like receptor 1 (Q6GTX8), leukocyte‐associated immunoglobulin‐like receptor 2 (Q6ISS4) and osteoclast‐associated immunoglobulin‐like receptor (Q8IYS5).

## Type XVII RTKs: ROS receptors

**Table:** 

Nomenclature	c‐ros oncogene 1, receptor tyrosine kinase
Common abreviation	ROS
HGNC, UniProt	*ROS1*, P08922
EC number	2.7.10.1

### Comments

crizotinib is a tyrosine kinase inhibitor, anti‐cancer drug targeting ALK and ROS1.

## Type XVIII RTKs: LMR family

### Overview

The LMR kinases are unusual amongst the RTKs in possessing a short extracellular domain and extended intracellular domain (hence the ‘Lemur’ name reflecting the long tail). A precise function for these receptors has yet to be defined, although LMR1 was identified as a potential marker of apoptosis [52], giving rise to the name AATYK (Apoptosis‐associated tyrosine kinase); while over‐expression induces differentiation in neuroblastoma cells [156].

**Table:** 

Nomenclature	apoptosis‐associated tyrosine kinase	lemur tyrosine kinase 2	lemur tyrosine kinase 3
Common abreviation	Lmr1	Lmr2	Lmr3
HGNC, UniProt	*AATK*, Q6ZMQ8	*LMTK2*, Q8IWU2	*LMTK3*, Q96Q04
EC number	2.7.11.1	2.7.11.1	2.7.11.1

### Comments

As yet no selective inhibitors of the LMR family have been described.

## Type XIX RTKs: Leukocyte tyrosine kinase (LTK) receptor family

### Overview

The LTK family appear to lack endogenous ligands. LTK is subject to tissue‐specific splice variation, which appears to generate products in distinct subcellular locations. ALK fusions created by gene translocations and rearrangements are associated with many types of cancer, including large cell lymphomas, inflammatory myofibrilastic tumours and non‐small cell lung cancer [120].

**Table:** 

Nomenclature	leukocyte receptor tyrosine kinase	anaplastic lymphoma receptor tyrosine kinase
Common abreviation	LTK	ALK
HGNC, UniProt	*LTK*, P29376	*ALK*, Q9UM73
EC number	2.7.10.1	2.7.10.1
Inhibitors	–	GSK‐1838705A (pIC_50_ 9.3) [161], compound 8e [PMID: 24432909] (pIC_50_ 9.1) [76], crizotinib (pIC_50_ 9) [35], NVP‐TAE684 (p*K*_d_ 9) [38], compound 25b [PMID: 22564207] (pIC_50_ 8.7) [59]
Selective inhibitors	–	ceritinib (pIC_50_ 9.7) [120]
Comments	–	crizotinib appears to be a selective ALK inhibitor acting on the tyrosine kinase activity [58]

## Type XX RTKs: STYK1

### Overview

Similar to the LMR RTK family, STYK1 has a truncated extracellular domain, but also displays a relatively short intracellular tail beyond the split kinase domain. STYK1 (also known as Novel Oncogene with Kinase‐domain, NOK) has been suggested to co‐localize with activated EGF receptor [43].

**Table:** 

Nomenclature	serine/threonine/tyrosine kinase 1
Common abreviation	STYK1
HGNC, UniProt	*STYK1*, Q6J9G0
EC number	2.7.10.2

### Comments

As yet, no selective inhibitors of STYK1 have been described.

## Receptor tyrosine phosphatases (RTP)

### Overview

Receptor tyrosine phosphatases (RTP) are cell‐surface proteins with a single TM region and intracellular phosphotyrosine phosphatase activity. Many family members exhibit constitutive activity in heterologous expression, dephosphorylating intracellular targets such as Src tyrosine kinase (SRC) to activate signalling cascades. Family members bind components of the extracellular matrix or cell‐surface proteins indicating a role in intercellular communication. Listed here are those family members with putative endogenous ligands.

**Table:** 

Nomenclature	RTP Type C	RTP Type D	RTP Type F	RTP Type G
HGNC, UniProt	*PTPRC*, P08575	*PTPRD*, P23468	*PTPRF*, P10586	*PTPRG*, P23470
Putative endogenous ligands	galectin‐1 (*LGALS1*, P09382) [188]	netrin‐G3 ligand (*LRRC4B*, Q9NT99) [96]	netrin‐G3 ligand (*LRRC4B*, Q9NT99) [96]	contactin‐3 (*CNTN3*, Q9P232), contactin‐4 (*CNTN4*, Q8IWV2), contactin‐5 (*CNTN5*, O94779), contactin‐6 (*CNTN6*, Q9UQ52) [16]

**Table:** 

Nomenclature	RTP Type K	RTP Type S	RTP Type Z1
HGNC, UniProt	*PTPRK*, Q15262	*PTPRS*, Q13332	*PTPRZ1*, P23471
Putative endogenous ligands	galectin‐3 (*LGALS3*, P17931), galectin‐3 binding protein (*LGALS3BP*, Q08380) [90]	chondroitin sulphate proteoglycan 3 (*NCAN*, O14594), netrin‐G3 ligand (*LRRC4B*, Q9NT99) [96, 166]	contactin‐1 (*CNTN1*, Q12860), pleiotrophin (*PTN*, C9JR52) (acts as a negative regulator) [16, 127]

### Further Reading

Böhmer F *et al*. (2013) Protein tyrosine phosphatase structure‐function relationships in regulation and pathogenesis. *FEBS J.* **280**: 413‐31 [PMID:22682070]

Dushek O *et al*. (2012) Non‐catalytic tyrosine‐phosphorylated receptors. *Immunol. Rev.* **250**: 258‐76 [PMID:23046135]

He R *et al*. (2013) Small molecule tools for functional interrogation of protein tyrosine phosphatases. *FEBS J.* **280**: 731‐50 [PMID:22816879]

Julien SG *et al*. (2011) Inside the human cancer tyrosine phosphatome. *Nat. Rev. Cancer* **11**: 35‐49 [PMID:21179176]

Mohebiany AN *et al*. (2013) Receptor‐type tyrosine phosphatase ligands: looking for the needle in the haystack. *FEBS J.* **280**: 388‐400 [PMID:22682003]

Sastry SK *et al*. (2011) Checks and balances: interplay of RTKs and PTPs in cancer progression. *Biochem. Pharmacol.* **82**: 435‐40 [PMID:21704606]

## Tumour necrosis factor (TNF) receptor family

### Overview

The TNF receptor superfamily (TNFRSF, provisional nomenclature) displays limited homology beyond an extracellular domain rich in cysteine residues and is activated by at least 18 different human homologues of TNF referred to as the TNF superfamily (TNFSF). Some homologues lacking transmembrane and cytoplasmic domains function as decoy receptors binding ligand without inducing cell signalling. Many of these receptors and ligands function as multimeric entities. Signalling through these receptors is complex and involves interaction with cytoplasmic adaptor proteins (such as TRADD and TRAF1). Several of these receptors contain cytoplasmic motifs known as ‘death domains’, which upon activation serve to recruit death domain‐ and death effector domain‐containing proteins crucial for the initiation of an apoptotic response. Additional signalling pathways include the regulation of the nuclear factor *κ*B or mitogen‐activated protein kinase pathways. Pharmacological manipulation of these receptors is mainly enacted through chelating the endogenous agonists with humanised monoclonal antibodies (*e.g*. Infliximab or adalimumab) or recombinant fusion proteins of IgG and soluble receptors (*e.g.* etanercept). Some mutated forms of TNF ligands are capable of selecting for different receptor subtypes.

**Table:** 

Nomenclature	tumor necrosis factor receptor 1	tumor necrosis factor receptor 2	lymphotoxin *β* receptor	OX40	CD40
Systematic nomenclature	TNFRSF1A	TNFRSF1B	TNFRSF3	TNFRSF4	TNFRSF5
Common abreviation	TNFR1	TNFR2	–	–	–
HGNC, UniProt	*TNFRSF1A*, P19438	*TNFRSF1B*, P20333	*LTBR*, P36941	*TNFRSF4*, P43489	*CD40*, P25942
Adaptor proteins	TRADD	TRAF1, TRAF2, TRAF5	TRAF3, TRAF4, TRAF5	TRAF1, TRAF2, TRAF3, TRAF5	TRAF1, TRAF2, TRAF3, TRAF5, TRAF6
Endogenous ligands	lymphotoxin‐*α* (*LTA*, P01374), tumour necrosis factormembrane form (*TNF*, P01375), tumour necrosis factorshed form (*TNF*, P01375)	lymphotoxin‐*α* (*LTA*, P01374), tumour necrosis factormembrane form (*TNF*, P01375)	LIGHT (*TNFSF14*, O43557), lymphotoxin *β*_2_*α*_1_ heterotrimer (*LTA* *LTB*, P01374 Q06643)	OX‐40 ligand (*TNFSF4*, P23510)	CD40 ligand (*CD40LG*, P29965)

**Table:** 

Nomenclature	Fas	decoy receptor 3	CD27	CD30	4‐1BB
Systematic nomenclature	TNFRSF6	TNFRSF6B	TNFRSF7	TNFRSF8	TNFRSF9
HGNC, UniProt	*FAS*, P25445	*TNFRSF6B*, O95407	*CD27*, P26842	*TNFRSF8*, P28908	*TNFRSF9*, Q07011
Adaptor proteins	FADD	–	TRAF2, SIVA	TRAF1, TRAF2, TRAF3, TRAF5	TRAF1, TRAF2, TRAF3
Endogenous ligands	Fas ligand (*FASLG*, P48023)	–	CD70 (*CD70*, P32970)	CD30 ligand (*TNFSF8*, P32971)	4‐1BB ligand (*TNFSF9*, P41273)
Antibodies	–	–	–	brentuximab vedotin (Inhibition)	–
Comments	–	Decoy receptor for LIGHT (*TNFSF14*, O43557), TL1A (*TNFSF15*, O95150) and Fas ligand (*FASLG*, P48023).	–	–	–

**Table:** 

Nomenclature	death receptor 4	death receptor 5	decoy receptor 1	decoy receptor 2	receptor activator of NF‐kappa B
Systematic nomenclature	TNFRSF10A	TNFRSF10B	TNFRSF10C	TNFRSF10D	TNFRSF11A
Common abreviation	DR4	DR5	–	–	RANK
HGNC, UniProt	*TNFRSF10A*, O00220	*TNFRSF10B*, O14763	*TNFRSF10C*, O14798	*TNFRSF10D*, Q9UBN6	*TNFRSF11A*, Q9Y6Q6
Adaptor proteins	FADD	FADD	–	–	TRAF1, TRAF2, TRAF3, TRAF5, TRAF6
Endogenous ligands	TRAIL (*TNFSF10*, P50591)	TRAIL (*TNFSF10*, P50591)	–	–	RANK ligand (*TNFSF11*, O14788)
Comments	–	–	Decoy receptor for TRAIL (*TNFSF10*, P50591).	Decoy receptor for TRAIL (*TNFSF10*, P50591).	–

**Table:** 

Nomenclature	osteoprotegerin	death receptor 3	TWEAK receptor	TACI
Systematic nomenclature	TNFRSF11B	TNFRSF25	TNFRSF12A	TNFRSF13B
Common abreviation	OPG	DR3	–	–
HGNC, UniProt	*TNFRSF11B*, O00300	*TNFRSF25*, Q93038	*TNFRSF12A*, Q9NP84	*TNFRSF13B*, O14836
Adaptor proteins	–	TRADD	TRAF1, TRAF2, TRAF3	TRAF2, TRAF5, TRAF6
Endogenous ligands	–	TL1A (*TNFSF15*, O95150)	TWEAK (*TNFSF12*, O43508)	APRIL (*TNFSF13*, O75888), BAFF (*TNFSF13B*, Q9Y275)
Comments	Acts as a decoy receptor for RANK ligand (*TNFSF11*, O14788) and possibly for TRAIL (*TNFSF10*, P50591).	–	–	–

**Table:** 

Nomenclature	BAFF receptor	herpes virus entry mediator	nerve growth factor receptor	B cell maturation antigen
Systematic nomenclature	TNFRSF13C	TNFRSF14	TNFRSF16	TNFRSF17
Common abreviation	BAFF‐R	HVEM	–	BCMA
HGNC, UniProt	*TNFRSF13C*, Q96RJ3	*TNFRSF14*, Q92956	*NGFR*, P08138	*TNFRSF17*, Q02223
Adaptor proteins	TRAF3	TRAF2, TRAF3, TRAF5	TRAF2, TRAF4, TRAF6	TRAF1, TRAF2, TRAF3, TRAF5, TRAF6
Endogenous ligands	BAFF (*TNFSF13B*, Q9Y275)	B and T lymphocyte attenuator (*BTLA*, Q7Z6A9), LIGHT (*TNFSF14*, O43557), lymphotoxin‐*α* (*LTA*, P01374)	BDNF (*BDNF*, P23560), NGF (*NGF*, P01138), neurotrophin‐3 (*NTF3*, P20783), neurotrophin‐4 (*NTF4*, P34130)	APRIL (*TNFSF13*, O75888), BAFF (*TNFSF13B*, Q9Y275)

**Table:** 

Nomenclature	glucocorticoid‐induced TNF receptor	toxicity and JNK inducer	RELT	death receptor 6
Systematic nomenclature	TNFRSF18	TNFRSF19	TNFRSF19L	TNFRSF21
Common abreviation	GITR	TAJ	–	DR6
HGNC, UniProt	*TNFRSF18*, Q9Y5U5	*TNFRSF19*, Q9NS68	*RELT*, Q969Z4	*TNFRSF21*, O75509
Adaptor proteins	TRAF1, TRAF2, TRAF3, SIVA	TRAF1, TRAF2, TRAF3, TRAF5	TRAF1	TRADD
Endogenous ligands	TL6 (*TNFSF18*, Q9UNG2)	lymphotoxin‐*α* (*LTA*, P01374)	–	–

**Table:** 

Nomenclature	TNFRSF22	TNFRSF23	ectodysplasin A2 isoform receptor	ectodysplasin 1, anhidrotic receptor
Systematic nomenclature	–	–	TNFRS27	–
HGNC, UniProt	–	–	*EDA2R*, Q9HAV5	*EDAR*, Q9UNE0
Adaptor proteins	–	–	TRAF1, TRAF3, TRAF6	TRAF1, TRAF2, TRAF3
Endogenous ligands	–	–	ectodysplasin A2 (*EDA*, Q92838) [204]	ectodysplasin A1 (*EDA*, Q92838) [204]

### Comments

TNFRSF1A is preferentially activated by the shed form of TNF ligand, whereas the membrane‐bound form of TNF serves to activate TNFRSF1A and TNFRSF1B equally. The neurotrophins nerve growth factor (NGF (*NGF*, P01138)), brain‐derived neurotrophic factor (BDNF (*BDNF*, P23560)), neurotrophin‐3 (*NTF3*, P20783) (NTF3) and neurotrophin‐4 (*NTF4*, P34130) (NTF4) are structurally unrelated to the TNF ligand superfamily but exert some of their actions through the “low affinity nerve growth factor receptor” (NGFR (TNFRSF16)) as well as through the TRK family of receptor tyrosine kinases. The endogenous ligands for EDAR and EDA2R are, respectively, the membrane (Q92838[1‐391]) and secreted (Q92838[160‐391]) isoforms of Ectodysplasin‐A (EDA, Q92838).

### Further Reading

Aggarwal BB. (2003) Signalling pathways of the TNF superfamily: a double‐edged sword. *Nat. Rev. Immunol.* **3**: 745‐56 [PMID:12949498]

Ashkenazi A. (2002) Targeting death and decoy receptors of the tumour‐necrosis factor superfamily. *Nat. Rev. Cancer* **2**: 420‐30 [PMID:12189384]

Mahmood Z *et al*. (2010) Death receptors: targets for cancer therapy. *Exp. Cell Res.* **316**: 887‐99 [PMID:20026107]

Rickert RC *et al*. (2011) Signaling by the tumor necrosis factor receptor superfamily in B‐cell biology and disease. *Immunol. Rev.* **244**: 115‐33 [PMID:22017435]

Tansey MG *et al*. (2009) The TNF superfamily in 2009: new pathways, new indications, and new drugs. *Drug Discov. Today* **14**: 1082‐8 [PMID:19837186]
